# A stereoselective and flexible synthesis to access both enantiomers of *N*-acetylgalactosamine and peracetylated *N*-acetylidosamine

**DOI:** 10.3762/bjoc.14.71

**Published:** 2018-04-13

**Authors:** Bettina Riedl, Walther Schmid

**Affiliations:** 1 University of Vienna, Institute of Organic Chemistry, Währinger Straße 38, A-1090 Vienna, Austria

**Keywords:** asymmetric synthesis, carbohydrates, *N*-acetylgalactosamine, *N*-acetylidosamine

## Abstract

Synthetic approaches towards *N*-acetylgalactosamine (GalNAc) have been attracting considerable interest since this compound is known for its pivotal role in cell–cell interaction and receptor induced cell signaling. Herein, we present a synthetic route in which two of the four stereogenic centers present in the target compound are derived from enantiopure tartaric acid being selectively converted to epoxy alcohols. The key step is the Pd-catalyzed, stereo- and regioselective epoxide opening and subsequent nucleophilic substitution of an azide functionality. This approach enables the synthesis of the naturally D- and unnaturally L-configured GalNAc, as well as both enantiomers of the largely unknown *N*-acetylidosamine (IdoNAc).

## Introduction

**Figure 1:**
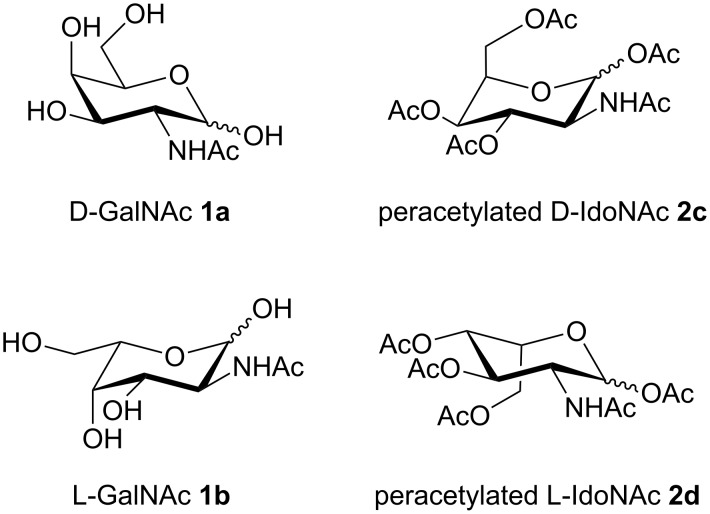
Four possible isomers reachable through the presented approach.

*N*-Acetylgalactosamine (GalNAc, Figure 1) and *N*-acetylidosamine (IdoNAc) belong to the group of 2-amino-2-deoxysugars, which can be found in a wide range of organisms as building blocks of, e.g., glycosaminoglycans, peptidoglycans or lipopolysaccharides [1]. As part of glycoproteins they are involved in numerous biological processes. On the one hand, these macromolecules influence the proper folding of newly synthesized proteins in the intracellular compartment of the endoplasmic reticulum (ER). On the other hand, glycoproteins serve as ligands for specific extracellular recognition processes toward, e.g., enzymes, lectins or antibodies [2]. In O-linked glycoproteins, also known as mucins, GalNAc becomes covalently α-linked to serine or threonine during post-translational modifications [3–5]. This glycoconjugate, known as T antigen nouvelle (Tn antigen), is further modified by the β(1→3) attachment of galactose leading to the Thomsen–Friedenreich (TF) antigen [6–7]. These two immunodeterminants are overexpressed on a high number of different cancer cells, located in the breast, lung, colon, liver, prostate and gastric tissues [8]. Furthermore, the TF-antigen region serves as the core region for the ABO and Lewis blood group determinants [9]. The high importance of GalNAc in diverse biological processes, together with its notable role in drug development, turns this compound into an interesting synthetic target. In contrast to the thoroughly investigated properties of GalNAc, other stereoisomers have not been probed for biological or pharmaceutical activity, yet. Highly flexible synthetic routes are required to access and probe the entire compound class of 2-amino-2-deoxysugars for further structure–activity relationship studies.

Several strategies for the synthesis of 2-amino sugars have been published so far. In one exemplary straightforward approach, GalNAc was prepared by inverting the stereogenic center at the C-4 position of *N*-acetylglucosamine (GlcNAc) [10]. However, the necessity of using a 2-amino sugar as starting material reduces the flexibility of this approach. Another strategy introduces the nitrogen at C-2 [11] via the 2-iodoxybenzoic acid-mediated generation of an oxazolidinone ring, selectively leading to C_2_–C_3_ *syn*-products [12]. A third approach features *E*-selective Julia olefination of (*R*)- or (*S*)-2,3-isopropylideneglyceraldehyde with a second chiral building block containing the amine function, followed by dihydroxylation [13–14]. Despite the compounds already available through these and other approaches, novel synthetic concepts to fill the remaining methodological gaps for accessing the missing isomers of 2-amino sugars, as well as new derivatives are still highly desired.

The here presented flexible approach towards GalNAc and IdoNAc from epoxythreitol **5** is a first step to answer this request. These molecules can be approached from tartaric acid (**3**), which already provides two of the four desired stereogenic centers. By extending compound **5** via a Horner–Wadsworth–Emmons (HWE) reaction [15] and subsequent stereoselective introduction of the corresponding nitrogen functionality, a route to four different 2-amino sugar stereoisomers has been elaborated.

## Results and Discussion

**Scheme 1:**
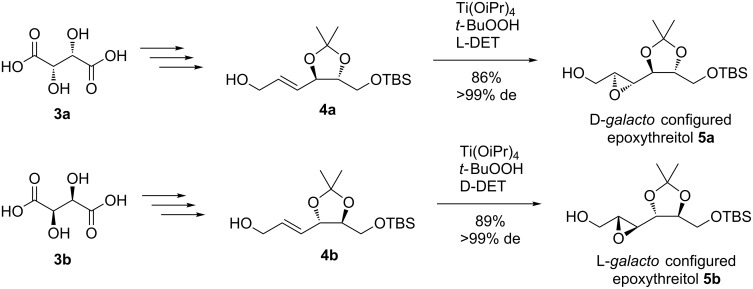
Sharpless epoxidation to gain D-*galacto-* **5a** and L-*galacto*-configured epoxythreitol **5b**.

A synthetic route was developed starting with both the D- and the L-epoxythreitol **5a** and **5b**. These isomers have been synthesized from D- (**3a**) and L-tartaric acid (**3b**), respectively, through an approach that has been published earlier by Iida et al. (Scheme 1) [16]. Here, tartaric acid predefines the configuration at C4 and C5 of the target compounds **5a** and **5b**. After the C4-chain of tartaric acid has been extended by two carbon atoms resulting in compounds **4a** and **4b**, two new stereocenters have been introduced by Sharpless epoxidation [17–18]. We applied this approach for the epoxidation of **4a** with L-diethyl tartrate (L-DET) directing the reaction towards the D-*galacto*-configured epoxythreitol **5a**. Alternatively, epoxidation of **4b** with D-DET was used to access L-*galacto*-configured epoxythreitol **5b**.

**Scheme 2:**
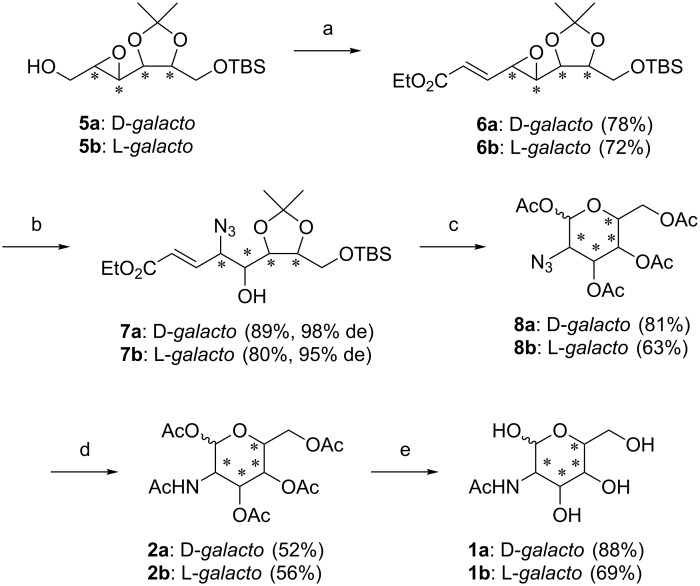
Reagents and conditions: a) i) (COCl)_2_, DMSO, Et_3_N, DCM, ii) triethyl phosphonoacetate, NaH, DCM; b) TMSN_3_, Pd(PPh_3_)_4_, EtOH; c) i) DOWEX H^+^, MeOH; ii) O_3_, dimethyl sulfide, DCM/MeOH; iii) Ac_2_O, DMAP, pyridine; d) H_2_, Pd/C, Ac_2_O; e) MeOH/H_2_O/Et_3_N (10:10:1).

**Scheme 3:**
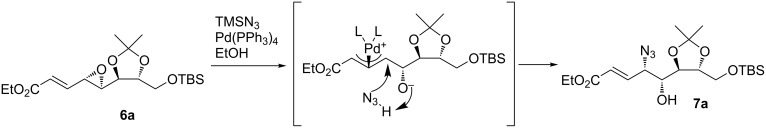
Proposed mechanism of the Pd-catalyzed azide substitution of **6a** in protic solvent.

A procedure by Miyashita et al. [19] for nucleophilic substitution of epoxides by an azide functionality was applied for the presented approach. Therefore, **5a** and **5b** had to be converted first to the unsaturated esters **6a** and **6b** (Scheme 2) [20–21]. These compounds were reacted with trimethylsilyl azide (TMSN_3_) under Pd^0^ catalysis. With this protocol, the azide was introduced under double inversion of the stereocenter on C4, resulting in the desired *cis*-selective epoxide opening and therefore, *galacto*-configured azido alcohols **7a** and **7b** (≥95% de) [22]. Initial limitations in reaction size and yield could have been overcome by switching the solvent from THF, as described in the Miyashita protocol, to EtOH. This improvement can be explained by a protonation of TMSN_3_ causing the in situ generation of highly reactive HN_3_ (Scheme 3). Additionally, no acidic work-up, as described by Miyashita et al., was necessary to gain the free alcohol on C5, which corroborates the proposed mechanism.

The protecting groups were cleaved by the use of acidic ion exchange (DOWEX H^+^). Subsequent ozonolysis caused the formation of the aldehyde by the oxidation of the methylene functionality. Consequently, intramolecular hemiacetal formation resulted immediately in the cyclization of the unprotected azido sugar, which was peracetylated to yield compounds **8a** and **8b**. The azide function was reduced with H_2_ and Pd/C in acetic anhydride to gain the fully acetylated amino sugars **2a** and **2b**. In the final step, the compounds were deprotected using a mixture of MeOH/H_2_O/Et_3_N to yield D-GalNAc (**1a**) and L-GalNAc (**1b**).

**Scheme 4:**
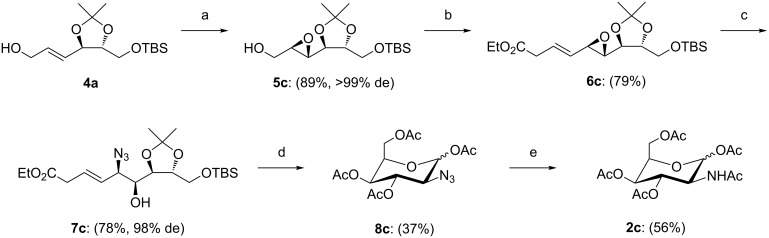
Approach towards peracetylated D-IdoNAc **2c**, reactions and conditions: a) Ti(OiPr)_4_, *t-*BuOOH, D-DET, DCM; b) i) (COCl)_2_, DMSO, Et_3_N, DCM, ii) triethyl phosphonoacetate, NaH, DCM; c) TMSN_3_, Pd(PPh_3_)_4_, EtOH; d) i) DOWEX H^+^, MeOH; ii) O_3_, dimethyl sulfide, DCM/MeOH; iii) Ac_2_O, DMAP, pyridine; e) H_2_, Pd/C, Ac_2_O.

As proof of concept, this approach was additionally adapted for the synthesis of peracetylated D-*N*-acetylidosamine **2c**. Therefore, the epoxidation of **4a** was performed with D-DET instead of L-DET, resulting in D-epoxythreitol **5c** (Scheme 4). Performing the presented, optimized reaction sequence resulted in the formation of peracetylated D-IdoNAc **2c**.

## Conclusion

The presented synthetic route was successfully used to prepare both, D- and L-GalNAc, from non-carbohydrate starting materials over 9 steps with overall yields of 22% and 12%, respectively. Our approach combines the Sharpless epoxidation and Pd-catalyzed azide substitution, both, highly stereoselective protocols resulting in an advantageous flexibility. Therefore, this route can also be adapted for the synthesis of the rarely known D- and L-IdoNAc. As a proof of concept, the synthesis of the peracetylated D-IdoNAc was performed (Scheme 4, 8 steps, 11%). Furthermore, this approach opens up new perspectives for additional modifications: *meso*-tartaric acid as possible starting material or different epoxide opening protocols, as well as, the selective isotopic labelling are only a few to mention. This convertible synthetic route brings us a step closer to access the whole library of 2-amino-2-deoxysugars, as well as a variety of derivatives, for further studies.

## Supporting Information

File 1 Experimental procedures, as well as ^1^H and ^13^C NMR spectra.
