# Supplementary material for: A stereoselective and flexible synthesis to access both enantiomers of N-acetylgalactosamine and peracetylated N-acetylidosamine
Source: Beilstein J Org Chem. 2018 Apr 13;14:856–60. doi: 10.3762/bjoc.14.71 (PMC5905266; doi:10.3762/bjoc.14.71)
Supplement: File 1 — Experimental procedures, as well as 1H and 13C NMR spectra. [file Beilstein_J_Org_Chem-14-856-s001.pdf]

Supporting Information

for

A stereoselective and flexible synthesis to access both enantiomers of *N*-acetylgalactosamine and peracetylated *N*-acetylidosamine

Bettina Riedl* and Walther Schmid

Address: University of Vienna, Institute of Organic Chemistry, Währinger Straße 38, A-1090 Vienna, Austria

Email: Bettina Riedl - bettina.riedl@univie.ac.at

* Corresponding author

Experimental procedures, as well as ^{1}H and ^{13}C NMR spectra

Table of contents

General information and methods S3

Experimental procedures S3

(2*R*,3*R*,4*S*,5*R*)-6-O-*tert*-Butyldimethylsilyl-2,3-epoxy-4,5-O-isopropylidene-hexan-1-ol (**5a**) S3

(2*S*,3*S*,4*R*,5*S*)-6-O-*tert*-Butyldimethylsilyl-2,3-epoxy-4,5-O-isopropylidene-hexan-1-ol (**5b**) S4

(2*S*,3*S*,4*S*,5*R*)-6-O-*tert*-Butyldimethylsilyl-2,3-epoxy-4,5-O-isopropylidene-hexan-1-ol (**5c**) S5

Ethyl (4*R*,5*R*,6*S*,7*R*)-8-O-*tert*-butyldimethylsilyl-4,5-epoxy-6,7-O-isopropylidene-(2*E*)-octenoate (**6a**) S5

Ethyl (4*S*,5*S*,6*R*,7*S*)-8-O-*tert*-butyldimethylsilyl-4,5-epoxy-6,7-O-isopropylidene-(2*E*)-octenoate (**6b**) S6

Ethyl (4*S*,5*S*,6*S*,7*R*)-8-O-*tert*-butyldimethylsilyl-4,5-epoxy-6,7-O-isopropylidene-(2*E*)-octenoate (**6c**) S7

Ethyl (4*S*,5*R*,6*R*,7*R*)-4-azido-8-O-*tert*-butyldimethylsilyl-6,7-O-isopropylidene-5-hydroxy-(2*E*)-octenoate (**7a**) S7

Ethyl (4*R*,5*S*,6*S*,7*S*)-4-azido-8-O-*tert*-butyldimethylsilyl-6,7-O-isopropylidene-5-hydroxy-(2*E*)-octenoate (**7b**) S8

Ethyl (4*R*,5*S*,6*R*,7*R*)-4-azido-8-O-*tert*-butyldimethylsilyl-6,7-O-isopropylidene-5-hydroxy-(2*E*)-octenoate (**7c**) S9

1,3,4,6-Tetra-O-acetyl-2-azido-2-deoxy-d-galactopyranose (**8a**) S9

1,3,4,6-Tetra-O-acetyl-2-azido-2-deoxy-l-galactopyranose (**8b**) S10

1,3,4,6-Tetra-O-acetyl-2-azido-2-deoxy-d-idopyranose (**8c**) S11

2-Acetamido-1,3,4,6-tetra-O-acetyl-2-deoxy-d-galactopyranose (**2a**) S12

2-Acetamido-1,3,4,6-tetra-O-acetyl-2-deoxy-l-galactopyranose (**2b**) S13

2-Acetamido-1,3,4,6-tetra-O-acetyl-2-deoxy-d-idopyranose (**2c**) S13

2-Acetamido-2-deoxy-d-galactopyranose (**1a**) S14

2-Acetamido-2-deoxy-l-galactopyranose (**1b**) S15

NMR spectra S16

General information and methods

NMR spectra were recorded on a Bruker Avance DRX 400 (100.13 MHz for ^{1}H, 100.61 MHz for ^{13}C) or a Bruker Avance III 600 (600.13 MHz for ^{1}H, 150.90 MHz for ^{13}C) spectrometer. Chemical shifts (δ) are given in parts per million [ppm]. Abbreviations for the multiplicities are as followed: singlet (s), doublet (d), triplet (t), quadruplet (q), multiplet (m). MS experiments were performed on a Finnigan MAT 900 spectrometer in ESI mode. IR spectra were verified on an ELMER FT-IR spectrometer. Optical rotations were measured on a Perkin Elmer Polarimeter 341. For flash chromatography, Merck silica gel 60 (0.004–0.063 mm) was used. DCM, acetone, MeOH, EtOH, heptane and ethyl acetate were distilled before use. Dry DCM was prepared by filtration through an aluminium oxide column and stored over molecular sieves 4 Å. Other solvents and chemicals were purchased in reagent grade.

Experimental procedures

(2*R*,3*R*,4*S*,5*R*)-6-*O*-*tert*-Butyldimethylsilyl-2,3-epoxy-4,5-*O*-isopropylidene-hexan-1-ol (5a)

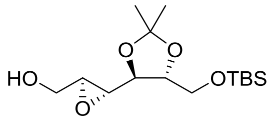

Titanium(IV) isopropoxide (2.7 mL, 9.26 mmol, 1.4 equiv), was dissolved under argon in dry DCM (50 mL), containing 4 Å molecular sieves (1.5 g), and cooled to −78 °C. Diethyl-(L)-tartrate (1.6 mL, 9.26 mmol, 1.4 equiv) was added and the mixture stirred for 15 min. Ethyl 6-*tert*-butyldimethylsilyloxy-(4*R*,5*R*)-isopropylidenedioxy-(3*E*)-hexenoate (**4a**, 2.00 g, 6.61 mmol, 1 equiv) in dry DCM (10 mL) was added dropwise followed by a 5.5 M solution of *tert*-butyl hydroperoxide in nonane (2.4 mL, 13.22 mmol, 2 equiv). The solution was stirred for 18 h at −20 °C and quenched subsequently by the addition of 10% solution of tartaric acid in water (25 mL). After stirring for an additional hour at rt, the mixture was filtrated through a Celite pad, the layers separated and the organic layer washed with sat. $NaHCO_3$ solution. Drying over $MgSO_4$, removing of the solvent under reduced pressure and purification by flash chromatography (heptane/ethyl acetate 4:1) yielded **5a** (1.86 g, 86%) as colorless oil: $[\alpha]^{20}_{D}$ = -16.19° (*c* 1.1, $CHCl_3$); ^{1}H NMR (600 MHz, $CDCl_3$) *δ* 4.01 – 3.92 (m, 3 H, H-2 and H-3 and H-6a), 3.79 – 3.71 (m, 2 H, H-1), 3.72 – 3.65 (m, 1 H, H-6b), 3.24 – 3.15 (m, 2 H, H-4 and H-5), 1.79 (dd, *J* = 7.5, 5.4 Hz, 1H, OH), 1.42 (s, 3 H, $C(CH_3)_2$), 1.41 (s, 3 H, $C(CH_3)_2$),

0.89 (s, 9 H, $C(CH_3)_3$), 0.07 (s, 6 H, $Si(CH_3)_2$); ^{13}C NMR (150 MHz, $CDCl_3$) δ 110.03 (C_q, $\underline{C}(CH_3)_2$), 79.12 (CH, C-2), 77.02 (CH, C-3), 63.56 (CH_2, C-1), 61.10 (CH_2, C-6), 56.14 (CH, C-5), 55.29 (CH, C-4), 27.05 (CH_3, $C(\underline{C}H_3)_2$), 26.89 (CH_3, $C(\underline{C}H_3)_2$), 26.03 (3 x CH_3, $C(\underline{C}H_3)_3$), 18.50 (C_q, $\underline{C}(CH_3)_3$), -5.22 (CH_3, $Si(CH_3)_2$), -5.30 (CH_3, $Si(CH_3)_2$); HRMS (ESI) *m/z* $[M + Na]^+$ calcd for $C_{15}H_{30}O_5SiNa$ 341.1755; found 341.1764.

(2*S*,3*S*,4*R*,5*S*)-6-*O*-*tert*-Butyldimethylsilyl-2,3-epoxy-4,5-*O*-isopropylidene-hexan-1-ol (5b)

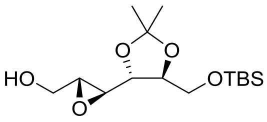

Titanium(IV) isopropoxide (2.7 mL, 9.26 mmol, 1.4 equiv), was dissolved under argon in dry DCM (50 mL) containing 4 Å molecular sieves (1.5 g) and cooled to −78 °C. Diethyl-(D)-tartrate (1.6 mL, 9.26 mmol, 1.4 equiv) was added and the mixture stirred for 15 min. Ethyl 6-*tert*-buthyldimethylsilyloxy-(4*S*,5*S*)-isopropylidenedioxy-(3*E*)-hexenoate (**4b**) (2.00 g, 6.61 mmol, 1 equiv) in dry DCM (10 mL) was added dropwise followed by a 5.5 M solution of *tert*-butyl hydroperoxide in nonane (2.4 mL, 13.22 mmol, 2 equiv). The solution was stirred for 18 h at −20 °C and quenched subsequently by the addition of 10 % solution of tartaric acid in water (25 mL). After stirring for an additional hour at rt, the mixture was filtrated through a Celite pad, the layers separated and the organic layer washed with sat. $NaHCO_3$ solution. Drying over $MgSO_4$, removing of the solvent under reduced pressure and purification by flash chromatography (heptane/ethyl acetate 4:1) yielded **5b** (1.87 g, 89%) as colorless oil: $[\alpha]^{20}_D$ = +15.69° (*c* 1.1, $CHCl_3$); ^1H NMR (400 MHz, $CDCl_3$) δ 4.02 (ddd, *J* = 7.8, 6.0, 4.0 Hz, 1 H, H-2), 3.96 (ddd, *J* = 12.8, 4.5, 2.4 Hz, 1 H, H-6a), 3.91 (dd, *J* = 7.9, 4.7 Hz, 1 H, H-3), 3.84 (dd, *J* = 10.6, 3.9 Hz, 1 H, H-1a), 3.72 (dd, *J* = 10.6, 6.0 Hz, 1 H, H-1b), 3.69 – 3.63 (m, 1 H, H-6b), 3.19 (dt, *J* = 3.8, 2.3 Hz, 1 H, H-5), 3.14 (dd, *J* = 4.7, 2.3 Hz, 1 H, H-4), 1.69 (dd, *J* = 7.6, 5.0 Hz, 1 H, OH), 1.40 (s, *J* = 0.7 Hz, 3 H, $C(CH_3)_2$), 1.39 (s, *J* = 0.7 Hz, 3 H, $C(CH_3)_2$), 0.89 (s, 9 H, $C(CH_3)_3$), 0.07 (s, 6 H, $Si(CH_3)_2$); ^{13}C NMR (100 MHz, $CDCl_3$) δ 109.99 (C_q, $\underline{C}(CH_3)_2$), 78.41 (CH, C-2), 78.19 (CH, C-3), 63.65 (CH_2, C-1), 60.88 (CH_2, C-6), 55.77 (CH, C-5), 54.98 (CH, C-4), 27.16 (CH_3, $C(\underline{C}H_3)_2$), 26.80 (CH_3, $C(\underline{C}H_3)_2$), 26.05 (3 x CH_3, $C(\underline{C}H_3)_3$), 18.51 (C_q, $\underline{C}(CH_3)_3$), -5.22 (CH_3, $Si(CH_3)_2$), -5.27 (CH_3, $Si(CH_3)_2$); HRMS (ESI) *m/z* $[M + Na]^+$ calcd for $C_{15}H_{30}O_5SiNa$ 341.1755; found 341.1758.

(2*S*,3*S*,4*S*,5*R*)-6-*O*-*tert*-Butyldimethylsilyl-2,3-epoxy-4,5-*O*-isopropylidene-hexan-1-ol (5c)

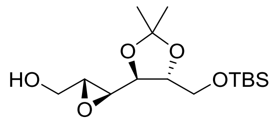

Compound **5c** was synthesized from **4a** (3.15 g, 10.41 mmol) according to the procedure for compound **5b**: yield 2.97 g (89%); $[\alpha]^{20}_{D}$ = +12.78° (*c* 1.6, $CHCl_3$); ^{1}H NMR (600 MHz, $CDCl_3$) *δ* 3.99 – 3.91 (m, 3H, H-2 and H-3 and H-6a), 3.79 – 3.72 (m, 2H, H-1), 3.67 (ddd, *J* = 12.8, 7.4, 4.0 Hz, 1H, H-6b), 3.19 (ddd, *J* = 7.7, 4.1, 2.2 Hz, 2H, H-4 and H-5), 1.79 (dd, *J* = 7.5, 5.4 Hz, 1H, OH), 1.41 (s, 3H, $C(CH_3)_2$), 1.40 (s, 3H, $C(CH_3)_2$), 0.89 (s, 9H, $C(CH_3)_3$), 0.07 (s, 6H, $Si(CH_3)_2$); ^{13}C NMR (150 MHz, $CDCl_3$) *δ* 110.02 (C_q), 79.11 (CH), 76.99 (CH), 63.53 (CH_2), 61.11 (CH_2), 56.17 (CH), 55.29 (CH), 27.03 (CH_3), 26.87 (CH_3), 26.02 (3 x CH_3, $C(\underline{C}H_3)_3$), 18.48 (C_q, $\underline{C}(CH_3)_3$), -5.23 (CH_3, $Si(CH_3)_2$), -5.28 (CH_3, $Si(CH_3)_2$); HRMS (ESI) *m/z* $[M + Na]^+$ calcd for $C_{15}H_{30}O_5SiNa$ 341.1755; found 341.1751.

Ethyl (4*R*,5*R*,6*S*,7*R*)-8-*O*-*tert*-butyldimethylsilyl-4,5-epoxy-6,7-*O*-isopropylidene-(2*E*)-octenoate (6a)

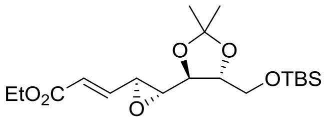

DMSO (2.9 mL, 41.33 mmol, 4 equiv) was added slowly to a solution of oxalyl chloride (1.8 mL, 20.66 mmol, 2 equiv) in dry DCM (35 mL) at −78 °C. After 15 min **5a** (3.29 g, 10.33 mmol, 1 equiv) in DCM (30 mL) was added dropwise and stirring continued for an additional hour. The reaction was quenched by the addition of Et_3N (8.6 mL, 61.99 mmol, 6 equiv) and allowed to warm up to rt over 16 h. The mixture was washed with sat. NH_4Cl solution and brine. The organic phase was dried over $MgSO_4$, solvents removed under reduced pressure and the crude aldehyde used without further purification.

NaH (10% in mineral oil, 496 mg, 12.40 mmol, 1.2 equiv) was dissolved in dry DCM (35 mL) under argon and cooled to 0 °C. Triethyl phosphonoacetate (2.7 mL, 12.40 mmol, 1.2 equiv) was added slowly and the mixture stirred for 1 h at room temperature. Subsequently, the intermediate, dissolved in dry DCM (20 mL), was added and the solution stirred for 16 h. The

reaction was quenched at 0 °C by the slow addition of water. The phases were separated and the aqueous phase extracted 3× with DCM. The organic phase was washed with brine and dried over $MgSO_4$. Removing of the solvents under reduced pressure and purification by flash chromatography (heptane/ethyl acetate 9:1) yielded **6a** (3.10 g, 78%) as a colorless oil: $[\alpha]^{20}_{D}$ = -7.57° (*c* 1.1, $CHCl_3$); ^{1}H NMR (600 MHz, $CDCl_3$) *δ* 6.67 (dd, *J* = 15.7, 7.1 Hz, 1 H, H-3), 6.15 (d, *J* = 15.7 Hz, 1 H, H-2), 4.19 (q, *J* = 7.1 Hz, 2 H, $OC\underline{H}_2CH_3$), 3.98 (dt, *J* = 9.2, 4.7 Hz, 1 H, H-7), 3.93 (dd, *J* = 7.4, 4.5 Hz, 1 H, H-6), 3.78 (dd, *J* = 10.7, 4.0 Hz, 1 H, H-8a), 3.73 (dd, *J* = 10.7, 5.3 Hz, 1 H, H-8b), 3.52 – 3.49 (m, 1 H, H-4), 3.10 (dd, *J* = 4.2, 1.3 Hz, 1 H, H-5), 1.41 (s, 3 H, $C(CH_3)_2$), 1.40 (s, 3 H, $C(CH_3)_2$), 1.28 (t, *J* = 7.2 Hz, 3 H, $OCH_2C\underline{H}_3$), 0.89 (s, 9 H, $C(CH_3)_3$), 0.07 (s, 6 H, $Si(CH_3)_2$); ^{13}C NMR (150 MHz, $CDCl_3$) *δ* 165.59 (C_q, C-1), 143.44 (CH, C-3), 124.64 (CH, C-2), 110.20 (C_q, $\underline{C}(CH_3)_2$), 79.02 (CH, C-7), 77.14 (CH, C-6), 63.47 (CH_2, C-8), 60.82 (CH, C-5), 60.80 (CH_2, $O\underline{C}H_2CH_3$), 54.12 (CH, C-4), 27.02 (CH_3, $C(\underline{C}H_3)_2$), 26.81 (CH_3, $C(\underline{C}H_3)_2$), 26.02 (3 x CH_3, $C(\underline{C}H_3)_3$), 18.48 (C_q, $\underline{C}(CH_3)_3$), 14.33 (CH_3, $OCH_2\underline{C}H_3$), -5.23 (CH_3, $Si(CH_3)_2$), -5.32 (CH_3, $Si(CH_3)_2$); HRMS (ESI) *m/z* $[M + Na]^+$ calcd for $C_{19}H_{34}O_6SiNa$ 409.2017; found 409.2016.

Ethyl (4*S*,5*S*,6*R*,7*S*)-8-*O*-*tert*-butyldimethylsilyl-4,5-epoxy-6,7-*O*-isopropylidene-(2*E*)-octenoate (6b)

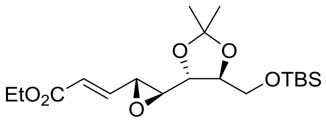

Compound **6b** was synthesized from **5b** (1.31 g, 4.11 mol) according to the procedure for compound **6a**: yield 1.15 g (72%); $[\alpha]^{20}_{D}$ = +6.80° (*c* 1.1, $CHCl_3$); ^{1}H NMR (600 MHz, $CDCl_3$) *δ* 6.67 (dd, *J* = 15.7, 7.1 Hz, 1 H. H-3), 6.15 (dd, *J* = 15.6, 0.8 Hz, 1 H, H-2), 4.20 (q, *J* = 7.1 Hz, 2 H, $OC\underline{H}_2CH_3$), 3.98 (ddd, *J* = 7.5, 5.3, 4.1 Hz, 1 H, H-7), 3.93 (dd, *J* = 7.5, 4.4 Hz, 1 H, H-6), 3.78 (dd, *J* = 10.7, 4.1 Hz, 1 H, H-8a), 3.74 (dd, *J* = 10.7, 5.4 Hz, 1 H, H-8b), 3.53 – 3.49 (m, 1 H, H-4), 3.11 (dd, *J* = 4.5, 2.0 Hz, 1 H, H-5), 1.41 (s, 3 H, $C(CH_3)_2$), 1.40 (s, 3 H, $C(CH_3)_2$), 1.28 (t, *J* = 7.1 Hz, 3 H, $OCH_2C\underline{H}_3$), 0.89 (s, 9 H, $C(CH_3)_3$), 0.07 (s, 6 H, $Si(CH_3)_2$); ^{13}C NMR (150 MHz, $CDCl_3$) *δ* 165.60 (C_q, C-1), 143.44 (CH, C-3), 124.65 (CH, C-2), 110.21 (C_q, $\underline{C}(CH_3)_2$), 79.03 (CH, C-7), 77.15 (CH, C-6), 63.49 (CH_2, C-8), 60.83 (CH, C-5), 60.81 (CH_2, $O\underline{C}H_2CH_3$), 54.13 (CH, C-4), 27.03 (CH_3, $C(CH_3)_2$), 26.82 (CH_3, $C(CH_3)_2$), 26.02 (3 x CH_3, $C(\underline{C}H_3)_3$), 18.49 (C_q, $\underline{C}(CH_3)_3$), 14.34 (CH_3, $OCH_2\underline{C}H_3$), -5.22 (CH_3, $Si(CH_3)_2$), -5.31 (CH_3, $Si(CH_3)_2$); HRMS (ESI) *m/z* $[M + Na]^+$ calcd for $C_{19}H_{34}O_6SiNa$ 409.2017; found 409.2025.

Ethyl (4*S*,5*S*,6*S*,7*R*)-8-*O*-*tert*-butyldimethylsilyl-4,5-epoxy-6,7-*O*-isopropylidene-(2*E*)-octenoate (6c)

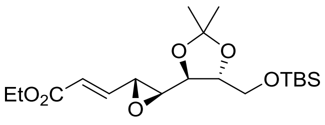

Compound **6c** was synthesized from **5c** (2.47 g, 7.76 mmol) according to the procedure for compound **6a**: yield 2.37 g (79%); $[\alpha]^{20}_D$ = +23.47° (*c* 1.0, $CHCl_3$); ^{1}H NMR (400 MHz, $CDCl_3$) *δ* 6.66 (ddd, *J* = 15.7, 7.3, 0.7 Hz, 1 H, H-3), 6.15 (dd, *J* = 15.6, 0.8 Hz, 1 H, H-2), 4.20 (q, *J* = 7.1 Hz, 2 H, $OC\underline{H}_2CH_3$), 4.05 (ddd, *J* = 7.6, 6.2, 3.9 Hz, 1 H, H-7), 3.99 (dd, *J* = 7.7, 3.9 Hz, 1 H, H-6), 3.85 (dd, *J* = 10.6, 3.9 Hz, 1 H, H-8a), 3.72 (dd, *J* = 10.6, 6.2 Hz, 1 H, H-8b), 3.53 (dt, *J* = 7.2, 1.3 Hz, 1 H, H-4), 3.05 (dd, *J* = 3.9, 2.0 Hz, 1 H, H-5), 1.39 (s, 6H, $C(CH_3)_2$), 1.29 (t, *J* = 7.1 Hz, 3 H, $OCH_2C\underline{H}_3$), 0.89 (s, 9 H, $C(CH_3)_3$), 0.08 (s, 6 H, $SI(CH_3)_2$); ^{13}C NMR (150 MHz, $CDCl_3$) δ 165.68 (C_q, C-1), 143.74 (CH, C-3), 124.60 (CH, C-2), 110.15 (C_q, $\underline{C}(CH_3)_2$), 78.09 (CH, C-7), 77.72 (CH, C-6), 63.57 (CH_2, C-8), 60.82 (CH_2, $O\underline{C}H_2CH_3$), 60.31 (CH, C-5), 53.80 (CH, C-4), 27.19 (CH_3, $C(\underline{C}H_3)_2$), 26.64 (CH_3, $C(\underline{C}H_3)_2$), 26.04 (3 x CH_3, $C(\underline{C}H_3)_3$), 18.50 (C_q, $\underline{C}(CH_3)_3$), 14.35 (CH_3, $OCH_2\underline{C}H_3$), -5.22 (CH_3, $Si(CH_3)_2$), -5.26 (CH_3, $Si(CH_3)_2$);HRMS (ESI) *m/z* $[M + Na]^+$ calcd for $C_{19}H_{34}O_6SiNa$ 409.2017; found 409.2034.

Ethyl (4*S*,5*R*,6*R*,7*R*)-4-azido-8-*O*-*tert*-butyldimethylsilyl-6,7-*O*-isopropylidene-5-hydroxy-(2*E*)-octenoate (7a)

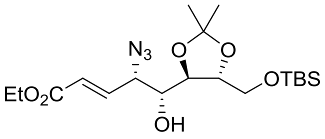

Compound **6a** (1.00 g, 2.59, 1 equiv) was dissolved in dry and degassed EtOH under argon. $Pd(PPh_3)_4$ (0.30 g, 0.26 mmol, 0.1 equiv) and trimethylsilyl azide (0.7 mL, 5.17 mmol, 2 equiv) were added and the mixture stirred for 4 h. Subsequently, the orange solid was filtered off. The solvents were removed under reduced pressure and the crude product purified by flash chromatography (heptane/ethyl acetate 5:1) to receive compound **7a** (0.99 g, 89%, 98% de) as a colorless oil: $[\alpha]^{20}_D$ = +3.78° (*c* 1.0, CH_2Cl_2); ^{1}H NMR (600 MHz, $CDCl_3$) *δ* 7.07 (dd, *J* = 15.7, 6.8 Hz, 1 H, H-3), 6.16 (dd, *J* = 15.7, 1.4 Hz, 1 H, H-2), 4.22 (q, *J* = 7.1 Hz, 2 H,

$OC\underline{H}_2CH_3$), 4.19 (d, *J* = 6.8 Hz, 1 H, H-5), 3.98 (dd, *J* = 9.6, 3.6 Hz, 1 H, H-8a), 3.93 –3.89 (m, 2 H, H-6 and H-7), 3.88 – 3.86 (m, 1 H, H-4), 3.68 (dt, *J* = 8.3, 2.5 Hz, 1 H, OH), 3.65 – 3.61 (m, 1 H, H-8b), 1.40 (s, 3 H, $C(CH_3)_2$), 1.38 (s, 3 H, $C(CH_3)_2$), 1.31 (t, *J* = 7.1 Hz, 3 H, $OCH_2C\underline{H}_3$), 0.91 (s, 9 H, $C(CH_3)_3$), 0.12 (s, 6 H, $Si(CH_3)_2$); ^{13}C NMR (150 MHz, $CDCl_3$) δ 165.86 (C_q, C-1), 142.22 (CH, C-3), 124.74 (CH, C-2), 109.77 (C_q, $\underline{C}(CH_3)_2$), 80.25 (CH, C-6), 80.04 (CH, C-7), 74.94 (CH, C-4), 64.39 (CH_2, C-8), 62.93 (CH, C-5), 60.85 (CH_2, $O\underline{C}H_2CH_3$), 26.93 (CH_3, $C(\underline{C}H_3)_2$), 26.91 (CH_3, $C(\underline{C}H_3)_2$), 25.99 (3 x CH_3, $C(\underline{C}H_3)_3$), 18.52 (C_q, $\underline{C}(CH_3)_3$), 14.37 (CH_3, $OCH_2\underline{C}H_3$), -5.41 (CH_3, $Si(CH_3)_2$), -5.42 (CH_3, $Si(CH_3)_2$); HRMS (ESI) *m/z* $[M + Na]^+$ calcd for $C_{19}H_{35}N_3O_6SiNa$ 452.2193; found 452.2195; (IR) v 3385 (OH), 2985 (CH), 2930 (CH), 2885 (CH), 2858 (CH), 2101 (N_3), 1722 (C=O) cm^{-1}.

Ethyl (4*R*,5*S*,6*S*,7*S*)-4-azido-8-*O*-*tert*-butyldimethylsilyl-6,7-*O*-isopropylidene-5-hydroxy-(2*E*)-octenoate (7b)

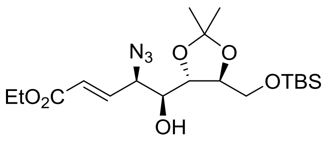

Compound **7b** was synthesized from **6b** (1.75 g, 4.53 mmol) according to the procedure for compound **7a**: yield 1.57 g (80%, 95% de); $[\alpha]^{20}_D$ = -2.76° (*c* 1.25, CH_2Cl_2); ^1H NMR (600 MHz, $CDCl_3$) δ 7.07 (dd, *J* = 15.7, 6.8 Hz, 1 H, H-3), 6.15 (dd, *J* = 15.7, 1.4 Hz, 1 H, H-2), 4.22 (q, *J* = 7.1 Hz, 2 H, $OC\underline{H}_2CH_3$), 4.20 – 4.17 (m, 1 H, H-5), 4.00 – 3.96 (m, 1 H, H-8a), 3.94 – 3.88 (m, 2 H, H-6 and H-7), 3.86 (dd, *J* = 2.7, 1.0 Hz, 1H, H-4), 3.68 (dt, *J* = 8.2, 2.4 Hz, 1 H, OH), 3.65 – 3.60 (m, 1 H, H-8b), 1.40 (s, 3 H, $C(CH_3)_2$), 1.38 (s, 3 H, $C(CH_3)_2$), 1.30 (t, *J* = 7.1 Hz, 3 H, $OCH_2C\underline{H}_3$), 0.91 (s, 9 H, $C(CH_3)_3$), 0.12 (s, 6 H, $Si(CH_3)_2$); ^{13}C NMR (150 MHz, $CDCl_3$) δ 165.84 (C_q, C-1), 142.21 (CH, C-3), 124.73 (CH, C-2), 109.76 (C_q, $\underline{C}(CH_3)_2$), 80.24 (CH, C-6), 80.04 (CH, C-7), 74.94 (CH, C-4), 64.39 (CH_2, C-8), 62.93 (CH, C-5), 60.83 (CH_2, $O\underline{C}H_2CH_3$), 26.93 (CH_3, $C(\underline{C}H_3)_2$), 26.90 (CH_3, $C(\underline{C}H_3)_2$), 25.99 (3 x CH_3, $C(\underline{C}H_3)_3$), 18.51 (C_q, $\underline{C}(CH_3)_2$), 14.37 (CH_3, $OCH_2\underline{C}H_3$), -5.42 (CH_3, $Si(CH_3)_2$), -5.43 (CH_3, $Si(CH_3)_2$); HRMS (ESI) *m/z* $[M + Na]^+$ calcd for $C_{19}H_{35}N_3O_6SiNa$ 452.2193; found 452.2186; (IR) v 3385 (OH), 2931 (CH), 2101 (N_3), 1722 (C=O) cm^{-1}.

Ethyl (4*R*,5*S*,6*R*,7*R*)-4-azido-8-*O*-*tert*-butyldimethylsilyl-6,7-*O*-isopropylidene-5-hydroxy-(2*E*)-octenoate (7c)

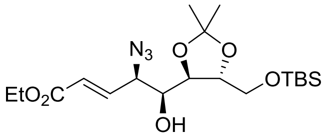

Compound **7c** was synthesized from **6c** (2.20 g, 5.69 mmol) according to the procedure for compound **7a**: yield 1.91 g (78%, 98% de); $[\alpha]^{20}_{D}$ = -14.64° (*c* 1.20, CH_2Cl_2); ^{1}H NMR (400 MHz, $CDCl_3$) *δ* 6.85 (dd, *J* = 15.6, 7.5 Hz, 1 H, H-3), 6.12 (dd, *J* = 15.6, 1.2 Hz, 1 H, H-2), 4.26 – 4.20 (m, 2 H, $OC\underline{H}_2CH_3$), 4.20 – 4.14 (m, 1 H, H-5), 4.10 (ddd, *J* = 7.9, 6.5, 3.9 Hz, 1 H, H-7), 3.98 (dd, *J* = 8.0, 2.0 Hz, 1 H, H-6), 3.83 (dd, *J* = 10.5, 3.9 Hz, 1 H, H-8a), 3.70 – 3.63 (m, 2 H, H-4 and H-8b), 2.62 (d, *J* = 8.7 Hz, 1 H, OH), 1.43 (s, 3 H, $C(CH_3)_2$), 1.39 (s, 3 H, $C(CH_3)_2$), 1.30 (t, *J* = 7.2 Hz, 3 H, $OCH_2C\underline{H}_3$), 0.88 (s, 9 H, $C(CH_3)_3$), 0.06 (s, 6 H, $Si(CH_3)_2$); ^{13}C NMR (150 MHz, $CDCl_3$) *δ* 165.48 (C_q, C-1), 140.87 (CH, C-3), 125.59 (CH, C-2), 109.98 (C_q, $\underline{C}(CH_3)_2$), 78.47 (CH, C-6), 77.05 (CH, C-7), 71.99 (CH, C-4), 65.74 (CH, C-5), 63.57 (CH_2, C-8), 60.92 (CH_2, $O\underline{C}H_2CH_3$), 27.22 (CH_3, $C(\underline{C}H_3)_2$), 27.03 (CH_3, $C(\underline{C}H_3)_2$), 25.97 (3 x CH_3, $C(\underline{C}H_3)_3$), 18.39 (C_q, $\underline{C}(CH_3)_3$), 14.33 (CH_3, $OCH_2\underline{C}H_3$), -5.37 (CH_3, $Si(CH_3)_2$), -5.40 (CH_3, $Si(CH_3)_2$); HRMS (ESI) *m/z* $[M + Na]^+$ calcd for $C_{19}H_{35}N_3O_6SiNa$ 452.2193; found 452.2188; (IR) v 3348 (OH), 2925 (CH), 2112 (N_3), 1738 (C=O) cm^{-1}.

1,3,4,6-Tetra-*O*-acetyl-2-azido-2-deoxy-D-galactopyranose (8a)

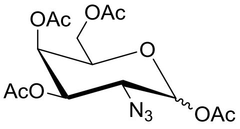

Compound **7a** (2.00 g, 4.66 mmol, 1 equiv) was dissolved in 10 mL MeOH/H_2O (4:1). After the addition of DOWEX H^+ (≈1 g), the mixture was heated to 40 °C for 16 h. DOWEX H^+ was filtered off and the solvents removed under reduced pressure. The crude product was used for the next step without further purification.

The solid was dissolved in DCM/MeOH (9:1) and ozone was purged through at −78 °C until the solution turned blue. Subsequently, oxygen was purged through until the blue color disappeared, again. Dimethyl sulfide (0.7 mL, 9.31 mmol, 2 equiv) was added and the

solution stirred overnight. The solution, containing partly precipitated product, was evaporated and the product used without further purification.

The solid was dissolved in 10 mL Ac_2O/pyridine (1:1) under argon. Dimethylaminopyridine (57 mg, 0.47 mmol, 0.1 equiv) was added and the mixture stirred overnight. The solvents were removed under reduced pressure and the crude product purified by flash chromatography (heptane/ethyl acetate 3:1) to receive compound **8a** (1.40 g, 81%, α-*pyr*/β-*pyr* 14:86): ^{1}H NMR (600 MHz, $CDCl_3$) *δ* α: 6.31 (d, *J* = 3.6 Hz, 1 H, H-1), 5.47 (dd, *J* = 3.2, 1.3 Hz, 1 H, H-4), 5.31 (dd, *J* = 11.1, 3.2 Hz, 1 H, H-3), 4.30 – 4.25 (m, 1 H, H-5), 4.11 – 4.04 (m, 2 H, H-6), 3.93 (dd, *J* = 11.1, 3.6 Hz, 1 H, H-2), 2.17 (s, 3 H; Ac), 2.16 (s, 3 H, Ac), 2.07 (s, 3 H, Ac), 2.03 (s, 3 H, Ac); β: 5.54 (d, *J* = 8.5 Hz, 1 H, H-1), 5.37 (dd, *J* = 3.3, 0.9 Hz, 1 H, H-4), 4.88 (dd, *J* = 10.8, 3.3 Hz, 1 H, H-3), 4.16 – 4.08 (m, 2 H, H-6), 4.00 (td, *J* = 6.7, 1.1 Hz, 1 H, H-5), 3.83 (dd, *J* = 10.8, 8.5 Hz, 1 H, H-2), 2.20 (s, 3 H, Ac), 2.16 (s, 3 H, Ac), 2.06 (s, 3 H, Ac), 2.03 (s, 3 H, Ac); ^{13}C NMR (150 MHz, $CDCl_3$) *δ* α: 170.48 (C_q, Ac), 170.11 (C_q, Ac), 169.99 (C_q, Ac), 168.85 (C_q, Ac), 90.52 (CH, C-1), 68.86 (CH, C-5), 68.77 (CH, C-3), 66.94 (CH, C-4), 61.21 (CH_2, C-6), 56.92 (CH, C-2), 21.08 (CH_3, Ac), 20.79 (3 x CH_3, Ac), 20.76 (CH_3, Ac); β: 170.46 (C_q, Ac), 170.07 (C_q, Ac), 169.75 (C_q, Ac), 168.70 (C_q, Ac), 92.96 (CH, C-1), 71.82 (CH, C-5), 71.40 (CH, C-3), 66.25 (CH, C-4), 61.05 (CH_2, C-6), 59.75 (CH, C-2), 21.03 (CH_3, Ac), 20.79 (CH_3, Ac), 20.74 (CH_3, Ac), 20.72 (CH_3, Ac); HRMS (ESI) *m/z* $[M + Na]^+$ calcd for $C_{14}H_{19}N_3O_9Na$ 396.1019; found 396.1016; (IR) v 2970 (CH), 2114 (N_3), 1747 (C=O) cm^{-1}.

1,3,4,6-Tetra-*O*-acetyl-2-azido-2-deoxy-L-galactopyranose (8b)

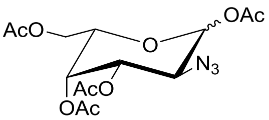

Compound **8b** was synthesized from **7b** (1.56 g, 3.63 mmol) according to the procedure for compound **8a**: yield 0.85 g (63%, α-*pyr*/β-*pyr* 19:81); ^{1}H NMR (600 MHz, $CDCl_3$) *δ* α: 6.31 (d, *J* = 3.6 Hz, 1 H, H-1), 5.48 – 5.45 (m, 1 H, H-4), 5.30 (ddd, *J* = 11.0, 3.3, 1.1 Hz, 1 H, H-3), 4.27 (t, *J* = 6.8 Hz, 1 H, H-5), 4.11 – 4.06 (m, 2 H, H-6), 3.93 (dd, *J* = 11.0, 3.6 Hz, 1 H, H-2), 2.17 (s, 3 H, Ac), 2.16 (s, 3 H, Ac), 2.06 (s, 3 H, Ac), 2.06 (s, 3 H, Ac); β: 5.54 (d, *J* = 8.5 Hz, 1 H, H-1), 5.38 – 5.35 (m, 1 H, H-4), 4.88 (dd, *J* = 10.8, 3.3 Hz, 1 H, H-3), 4.15 – 4.09 (m, 2 H, H-6), 4.00 (td, *J* = 6.7, 1.2 Hz, 1 H, H-5), 3.83 (ddd, *J* = 10.9, 8.5, 1.1 Hz, 1 H, H-2), 2.19 (s, 3 H, Ac), 2.16 (s, 3 H, Ac), 2.05 (s, 3 H, Ac), 2.03 (s, 3 H, Ac); ^{13}C NMR (150 MHz, $CDCl_3$)

δ α: 170.11 (C_q, Ac), 169.99 (C_q, Ac), 169.75 (C_q, Ac), 168.85 (C_q, Ac), 90.51 (CH, C-1), 68.86 (CH, C-5), 68.75 (CH, C-3), 66.94 (CH, C-4), 61.20 (CH_2, C-6), 56.91 (CH, C-2), 21.18 (CH_3, Ac), 20.06 (CH_3, Ac), 20.77 (CH_3, Ac), 20.72 (CH_3, Ac); β: 170.47 (C_q, Ac), 170.07 (C_q, Ac), 169.75 (C_q, Ac), 168.70 (C_q, Ac), 92.95 (CH, C-1), 71.81 (CH, C-5), 71.39 (CH, C-3), 66.26 (CH, C-4), 61.05 (CH_2, C-6), 59.74 (CH, C-2), 21.01 (CH_3, Ac), 20.77 (CH_3, Ac), 20.72 (CH_3, Ac), 20.70 (CH_3, Ac); HRMS (ESI) *m/z* $[M + Na]^+$ calcd for $C_{14}H_{19}N_3O_9SiNa$ 396.1019; found 396.1013; (IR) v 2969 (CH), 2113 (N_3), 1744 (C=O) cm^{-1}.

1,3,4,6-Tetra-*O*-acetyl-2-azido-2-deoxy-D-idopyranose (8c)

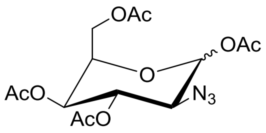

Compound **8c** was synthesized from **7c** (0.10 g, 0.23 mmol) according to the procedure for compound **8a**: yield 32 mg (37%, α-*pyr*/β-*pyr*/α-*fur* 50:31:19); ^1H NMR (600 MHz, $CDCl_3$) *δ* α-*pyr*: 6.00 (d, *J* = 4.4 Hz, 1 H, H-1), 5.12 (dd, *J* = 6.4, 5.2 Hz, 1 H, H-3), 5.04 (dd, *J* = 5.2, 3.6 Hz, 1 H, H-4), 4.45 (ddd, *J* = 6.6, 5.2, 3.6 Hz, 1 H, H-5), 4.27 – 4.21 (m, 2 H, H-6), 3.65 (dd, *J* = 6.4, 4.4 Hz, 1 H, H-2), 2.12 (s, 3 H, Ac), 2.11 (s, 3 H, Ac), 2.11 (s, 3 H, Ac), 2.07 (s, 3 H, Ac); β-*pyr*: 6.06 (d, *J* = 2.4 Hz, 1 H, H-1), 5.25 (t, *J* = 4.6 Hz, 1 H, H-3), 4.87 (dd, *J* = 4.5, 3.0 Hz, 1 H, H-4), 4.35 (ddd, *J* = 7.2, 5.6, 3.1 Hz, 1 H, H-5), 4.19 – 4.13 (m, 2 H, H-6), 3.57 (dd, *J* = 4.6, 2.4 Hz, 1 H, H-2), 2.18 (s, 3 H, Ac), 2.13 (s, 3 H, Ac), 2.13 (s, 3 H, Ac), 2.04 (s, 3 H, Ac); α-*fur*: 6.02 (d, *J* = 1.8 Hz, 1 H, H-1), 5.32 (td, *J* = 6.3, 3.8 Hz, 1 H, H-3), 5.17 (dd, *J* = 6.0, 3.2 Hz, 1 H, H-4), 4.52 (t, *J* = 6.2 Hz, 1 H, H-2), 4.29 (dd, *J* = 12.0, 3.8 Hz, 1H, H-6a), 4.16 – 4.14 (m, 1H, H-5), 4.01 (dd, *J* = 12.0, 6.4 Hz, 1H, H-6b), 2.13 (s, 3 H, Ac), 2.11 (s, 3 H, Ac), 2.10 (s, 3 H, Ac), 2.03 (s, 3 H, Ac); ^{13}C NMR (150 MHz, $CDCl_3$) *δ* α-*pyr*: 170.47 (C_q, Ac), 169.94 (C_q, Ac), 169.31 (C_q, Ac), 168.72 (C_q, Ac), 91.43 (CH, C-1), 68.94 (CH, C-3), 67.92 (CH, C-5), 67.63 (CH, C-4), 61.65 (CH_2, C-6), 58.27 (CH, C-2), 21.00 (CH_3, Ac), 20.85 (2 x CH_3, Ac), 20.69 (CH_3, Ac); β-*pyr*: 170.56 (C_q, Ac), 169.80 (C_q, Ac), 168.85 (C_q, Ac), 168.69 (C_q, Ac), 91.15 (CH, C-1), 72.29 (CH, C-5), 68.17 (CH, C-3), 66.04 (CH, C-4), 62.13 (CH_2, C-6), 57.49 (CH, C-2), 20.98 (CH_3, Ac), 20.89 (CH_3, Ac), 20.80 (CH_3, Ac), 20.71 (CH_3, Ac); α-*fur*: 170.53 (C_q, Ac), 170.10 (C_q, Ac), 169.94 (C_q, Ac), 169.58 (C_q, Ac), 98.68 (CH, C-1), 78.96 (CH, C-2), 75.09 (CH, C-4), 69.27 (CH, C-3 or C-5), 69.19 (CH, C-3 or C-5), 62.58 (CH_2, C-6), 21.13 (CH_3, Ac), 21.12 (CH_3, Ac), 20.78 (CH_3, Ac), 20.76 (CH_3, Ac); HRMS (ESI)

m/z [M + Na]$^+$ calcd for $C_{14}H_{19}N_3O_9SiNa$ 396.1019; found 396.1009; (IR) ν 2969 (CH), 2113 (N_3), 1744 (C=O) cm^{-1}.

2-Acetamido-1,3,4,6-tetra-*O*-acetyl-2-deoxy-D-galactopyranose (2a)

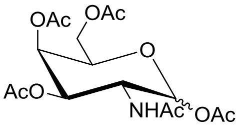

8a (200 mg, 0.54 mmol, 1 equiv) was dissolved in 3 mL acetic anhydride. 20 mg Pd/C was added and a H_2-ballon attached to the flask. The mixture was stirred for 16 h before acetic anhydride was removed under reduced pressure. The mixture was dissolved in warm EtOH/H_2O (1:1) and filtered through a Celite pad. The solvents were removed under reduced pressure and the crude product purified by flash chromatography (DCM/ethyl acetate/MeOH 7/2.5/0.5) to receive compound **2a** (108 mg, 52%, α-*pyr*/β-*pyr* 26:74) as a colorless solid: ^{1}H NMR (600 MHz, $CDCl_3$) *δ* α: 6.21 (d, *J* = 3.6 Hz, 1 H, H-1), 5.44 (d, *J* = 9.0 Hz, 1 H, NH), 5.42 (dd, *J* = 3.1, 1.2 Hz, 1 H, H-4), 5.22 (dd, *J* = 11.6, 3.1 Hz, 1 H, H-3), 4.72 (ddd, *J* = 11.6, 9.2, 3.6 Hz, 1 H, H-2), 4.25 – 4.22 (m, 1 H, H-5), 4.12 – 4.04 (m, 2 H, H-6), 2.17 (s, 6 H, Ac), 2.03 (s, 6 H, Ac), 1.95 (s, 3 H, Ac); β: 5.69 (d, *J* = 8.9 Hz, 1 H, H-1), 5.49 (d, *J* = 9.6 Hz, 1 H, NH), 5.37 (dd, *J* = 3.4, 1.2 Hz, 1 H, H-4), 5.08 (dd, *J* = 11.3, 3.4 Hz, 1 H, H-3), 4.44 (dt, *J* = 11.3, 9.3 Hz, 1 H, H-2), 4.18 – 4.08 (m, 2 H, H-6), 4.01 (td, *J* = 6.5, 1.2 Hz, 1 H, H-5), 2.16 (s, 3 H, Ac), 2.12 (s, 3 H, Ac), 2.04 (s, 3 H, Ac), 2.01 (s, 3 H, Ac), 1.93 (s, 3 H, Ac); ^{13}C NMR (150 MHz, $CDCl_3$) *δ* α: 171.38 (C_q, Ac), 170.56 (C_q, Ac), 170.38 (C_q, Ac), 170.21 (C_q, Ac), 168.94 (C_q, Ac), 91.49 (CH, C-1), 68.70 (CH, C-5), 67.97 (CH, C-3), 66.84 (CH, C-4), 61.45 (CH_2, C-6), 47.14 (CH, C-2), 23.34 (CH_3, Ac), 21.12 (CH_3, Ac), 20.91 (CH_3, Ac), 20.84 (CH_3, Ac), 20.82 (CH_3, Ac); β: 170.90 (C_q, Ac), 170.56 (C_q, Ac), 170.43 (C_q, Ac), 170.32 (C_q, Ac), 169.72 (C_q, Ac), 93.18 (CH, C-1), 72.00 (CH, C-5), 70.46 (CH, C-3), 66.48 (CH,C-4), 61.45 (CH_2, C-6), 49.93 (CH, C-2), 23.46 (CH_3, Ac), 21.05 (CH_3, Ac), 20.82 (2 x CH_3, Ac), 20.80 (CH_3, Ac); HRMS (ESI) *m/z* [M + Na]$^+$ calcd for $C_{16}H_{23}NO_{10}Na$ 412.1220; found 412.1223.

2-Acetamido-1,3,4,6-tetra-*O*-acetyl-2-deoxy-L-galactopyranose (2b)

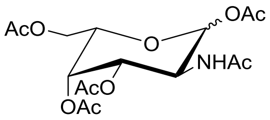

Compound **2b** was synthesized from **8b** (0.20 g, 0.54 mmol) according to the procedure for compound **2a**: yield 0.11 g (56%, α-*pyr*/β-*pyr* 57:43); ^{1}H NMR (600 MHz, $CDCl_3$) δ α: α: 6.21 (d, J = 3.6 Hz, 1 H, H-1), 5.46 (d, J = 9.2 Hz, 1 H, NH), 5.42 (dd, J = 3.4, 1.3 Hz, 1 H, H-4), 5.21 (dd, J = 11.5, 3.3 Hz, 1 H, H-3), 4.72 (ddd, J = 11.6, 9.2, 3.7 Hz, 1 H, H-2), 4.23 (td, J = 6.8, 1.4 Hz, 1 H, H-5), 4.11 (dd, J = 11.3, 6.7 Hz, 2 H, H-6), 2.17 (s, 6H, Ac), 2.03 (s, 6 H, Ac), 1.95 (s, 3 H, Ac); β: 5.70 (d, J = 8.8 Hz, 1 H, H-1), 5.53 (d, J = 9.6 Hz, 1 H, NH), 5.37 (dd, J = 3.4, 1.1 Hz, 1 H, H-4), 5.08 (dd, J = 11.3, 3.4 Hz, 1 H, H-3), 4.44 (dt, J = 11.3, 9.2 Hz, 1 H, H-2), 4.16 (dd, J = 11.4, 6.6 Hz, 1 H, H-6a), 4.06 (dd, J = 11.3, 6.5 Hz, 1 H, H-6b), 4.02 (td, J = 6.5, 1.2 Hz, 1 H, H-5), 2.16 (s, 3 H, Ac), 2.12 (s, 3 H, Ac), 2.04 (s, 3 H, Ac), 2.01 (s, 3 H, Ac), 1.94 (s, 3 H, Ac); ^{13}C NMR (150 MHz, $CDCl_3$) δ α: 171.35 (C_q, Ac), 170.54 (C_q, Ac), 170.37 (C_q, Ac), 170.21 (C_q, Ac), 168.94 (C_q, Ac), 91.49 (CH, C-1), 68.68 (CH, C-5), 67.96 (CH, C-3), 66.83 (CH, C-4), 61.43 (CH_2, C-6), 47.12 (CH, C-2), 23.32 (CH_3, Ac), 21.11 (CH_3, Ac), 20.90 (CH_3, Ac), 20.79 (2 x CH_3, Ac); β: 170.87 (C_q, Ac), 170.53 (C_q, Ac), 170.44 (C_q, Ac), 170.37 (C_q, Ac), 169.70 (C_q, Ac), 93.17 (CH, C-1), 71.96 (CH, C-5), 70.46 (CH, C-3), 66.47 (CH, C), 61.45 (CH_2, C-6), 49.88 (CH, C-2), 23.44 (CH_3, Ac), 21.04 (CH_3, Ac), 20.82 (2 x CH_3, Ac), 20.79 (CH_3, Ac); HRMS (ESI) *m/z* $[M + Na]^+$ calcd for $C_{16}H_{23}NO_{10}Na$ 412.1220; found 412.1217.

2-Acetamido-1,3,4,6-tetra-*O*-acetyl-2-deoxy-D-idopyranose (2c)

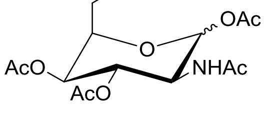

Compound **2c** was synthesized from **8c** (30 mg, 0.08 mmol) according to the procedure for compound **2a**: yield 18 mg (56%, α-*pyr*/β-*pyr*/α-*fur* 49:33:18); ^{1}H NMR (600 MHz, $CDCl_3$) δ α-*pyr*: 6.11 (d, J = 9.8 Hz, 1 H, NH), 5.93 (s, 1 H, H-1), 5.04 (t, J = 2.8 Hz, 1 H, H-4), 4.87 (t, J = 3.6 Hz, 1 H, H-3), 4.48 (td, J = 6.6, 2.1 Hz, 1 H, H-5), 4.31 (dt, J = 10.0, 2.9 Hz, 1 H, H-2), 4.11 (t, J = 6.2 Hz, 2 H, H-6), 2.14 (s, 3 H, Ac), 2.11 (s, 3 H, Ac), 2.10 (s, 3 H, Ac), 2.09 (s, 3

H, Ac), 2.04 (s, 3 H, Ac); β-*pyr*: 6.06 (d, J = 9.7 Hz, 1 H, NH), 6.01 (d, J = 2.4 Hz, 1 H, H-1), 5.07 (t, J = 4.2 Hz, 1 H, H-4), 4.98 (t, J = 3.3 Hz, 1 H, H-3), 4.39 – 4.33 (m, 2 H, H-2 and H-5), 4.18 – 4.13 (m, 2 H, H-6), 2.12 (s, 3 H, Ac), 2.11 (s, 3 H, Ac), 2.04 (s, 3 H, Ac), 2.02 (s, 3 H, Ac), 1.99 (s, 3 H, Ac); α-*fur*: 6.27 (d, J = 8.2 Hz, 1 H, NH), 6.02 (d, J = 3.2 Hz, 1 H, H-1), 5.32 (dd, J = 6.9, 5.4 Hz, 1 H, H-3), 5.25 (dt, J = 7.1, 4.8 Hz, 1 H, H-5), 4.61 (ddd, J = 8.3, 5.4, 3.2 Hz, 1 H, H-2), 4.55 (dd, J = 6.9, 4.8 Hz, 1 H, H-4), 4.26 (dd, J = 11.9, 4.1 Hz, 1 H, H-6a), 4.05 (dd, J = 11.9, 7.1 Hz, 1 H, H-6b), 2.14 (s, 3 H, Ac), 2.10 (s, 3 H, Ac), 2.07 (s, 3 H, Ac), 2.04 (s, 3 H, Ac), 1.98 (s, 3 H, Ac); ^{13}C NMR (150 MHz, $CDCl_3$) δ α-pyr: 170.59 (C_q, Ac), 169.71 (C_q, Ac), 169.04 (C_q, Ac), 168.68 (C_q, Ac), 168.66 (CH, Ac), 92.06 (CH, C-1), 67.36 (CH, C-3), 66.49 (CH, C-4), 66.04 (CH, C-5), 61.72 (CH_2, C-6), 46.32 (CH, C-2), 23.35 (CH_3, Ac), 20.99 (CH_3, Ac), 20.89 (CH_3, Ac), 20.84 (CH_3, Ac), 20.81 (CH_3, Ac); β-pyr: 170.34 (C_q, Ac), 170.17 (C_q, Ac), 170.02 (C_q, Ac), 168.76 (C_q, Ac), 168.57 (C_q, Ac), 90.80 (CH, C-1), 72.52 (CH, C-5), 68.48 (CH, C-4), 66.23 (CH, C-3), 61.94 (CH_2, C-6), 47.85 (CH, C-2), 23.35 (CH_3, Ac), 21.01 (CH_3, Ac), 20.91 (CH_3, Ac), 20.80 (CH_3, Ac), 20.74 (CH_3, Ac); α-fur: 170.62 (C_q, Ac), 170.55 (C_q, Ac), 170.36 (C_q, Ac), 169.89 (C_q, Ac), 168.93 (C_q, Ac), 98.76 (CH, C-1), 78.29 (CH, C-4), 75.05 (CH, C-3), 69.06 (CH, C-5), 62.70 (CH_2, C-6), 59.74 (CH, C-2), 23.18 (CH_3, Ac), 21.23 (CH_3, Ac), 21.16 (CH_3, Ac), 21.03 (CH_3, Ac), 20.87 (CH_3, Ac); HRMS (ESI) *m/z* $[M + Na]^+$ calcd for $C_{16}H_{23}NO_{10}Na$ 412.1220; found 412.1217.

2-Acetamido-2-deoxy-D-galactopyranose (1a)

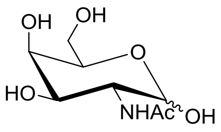

2a (115 mg, 0.30 mmol, 1 equiv) was dissolved in 3 mL MeOH/H_2O/Et_3N (10:10:1) and stirred for 16 h. The solvents were removed under reduced pressure and the crude product was recrystallized from MeOH/Et_2O to receive compound **1a** (57 mg, 88%, α-*pyr*/β-*pyr* 56:44) as a colorless solid: $[\alpha]^{20}_D$ = +80.0° (*c* 1.0, H_2O); ^{1}H NMR (600 MHz, D_2O) δ α: 5.20 (d, J = 3.7 Hz, 1 H, H-1), 4.12 – 4.06 (m, 2 H, H-2 and H-5), 3.97 (d, J = 2.7 Hz, 1 H, H-4), 3.91 (t, J = 2.7 Hz, 1 H, H-3), 3.77 – 3.69 (m, 2 H, H-6), 2.02 (s, 3 H, Ac); β: 4.62 (d, J = 8.4 Hz, 1 H, H-1), 3.88 (d, J = 3.2 Hz, 1 H, H-4), 3.85 (dd, J = 10.8, 8.5 Hz, 1 H, H-2), 3.79 – 3.69 (m, 3 H, H-3 and H-6), 3.69 – 3.65 (m, 1H, H-5), 2.02 (s, 3 H, Ac); ^{13}C NMR (150 MHz, H_2O) δ α: 175.45 (C_q, Ac), 91.73 (CH, C-1), 71.28 (CH, C-5), 69.31 (CH, C-4), 68.12 (CH, C-3), 61.97 (CH_2, C-6), 51.00 (CH, C-2), 22.70 (CH_3, Ac); β: 175.72 (C_q, Ac), 96.14 (CH, C-1), 75.93

(CH, C-5), 71.87 (CH, C-3), 68.59 (CH, C-4), 61.74 (CH_2, C-6), 54.39 (CH, C-2), 22.95 (CH_3, Ac); HRMS (ESI) *m/z* $[M + Na]^+$ calcd for $C_8H_{15}NO_6Na$ 244.0797; found 244.0793.

2-Acetamido-2-deoxy-L-galactopyranose (1b)

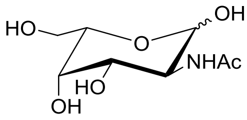

Compound **1b** was synthesized from **2b** (31 mg, 0.08 mmol) according to the procedure for compound **1a**: yield 16 mg (69%, α-*pyr*/β-*pyr* 68:32); $[\alpha]^{20}_D$ = -71.5° (*c* 1.0, H_2O); ^1H NMR (600 MHz, D_2O) δ α: 5.23 (d, *J* = 3.8 Hz, 1 H, H-1), 4.15 – 4.08 (m, 2 H, H-2 and H-5), 4.01 – 3.97 (m, 1 H, H-4), 3.93 (t, *J* = 3.4 Hz, 1 H, H-3), 3.74 (d, *J* = 6.1 Hz, 2 H, H-6), 2.05 (s, 3 H, Ac); β: 4.64 (d, *J* = 8.4 Hz, 1 H, H-1), 3.91 (d, *J* = 3.2 Hz, 1 H, H-4), 3.87 (dd, *J* = 10.9, 8.5 Hz, 1 H, H-2), 3.81 – 3.71 (m, 3 H, H-3 and H-6), 3.71 – 3.66 (m, 1H, H-5), 2.04 (s, 3 H, Ac); ^{13}C NMR (150 MHz, H_2O) δ α: 175.44 (C_q, Ac), 91.74 (CH, C-1), 71.28 (CH, C-5), 69.32 (CH, C-4), 68.14 (CH, C-3), 61.98 (CH_2, C-6), 51.01 (CH, C-2), 22.72 (CH_3, Ac); β: 175.72 (C_q, Ac), 96.16 (CH, C-1), 75.93 (CH, C-5), 71.88 (CH, C-3), 68.60 (CH, C-4), 61.75 (CH_2, C-6), 54.41 (CH, C-2), 22.97 (CH_3, Ac); HRMS (ESI) *m/z* $[M + Na]^+$ calcd for $C_8H_{15}NO_6Na$ 244.0797; found 244.0788.

NMR spectra

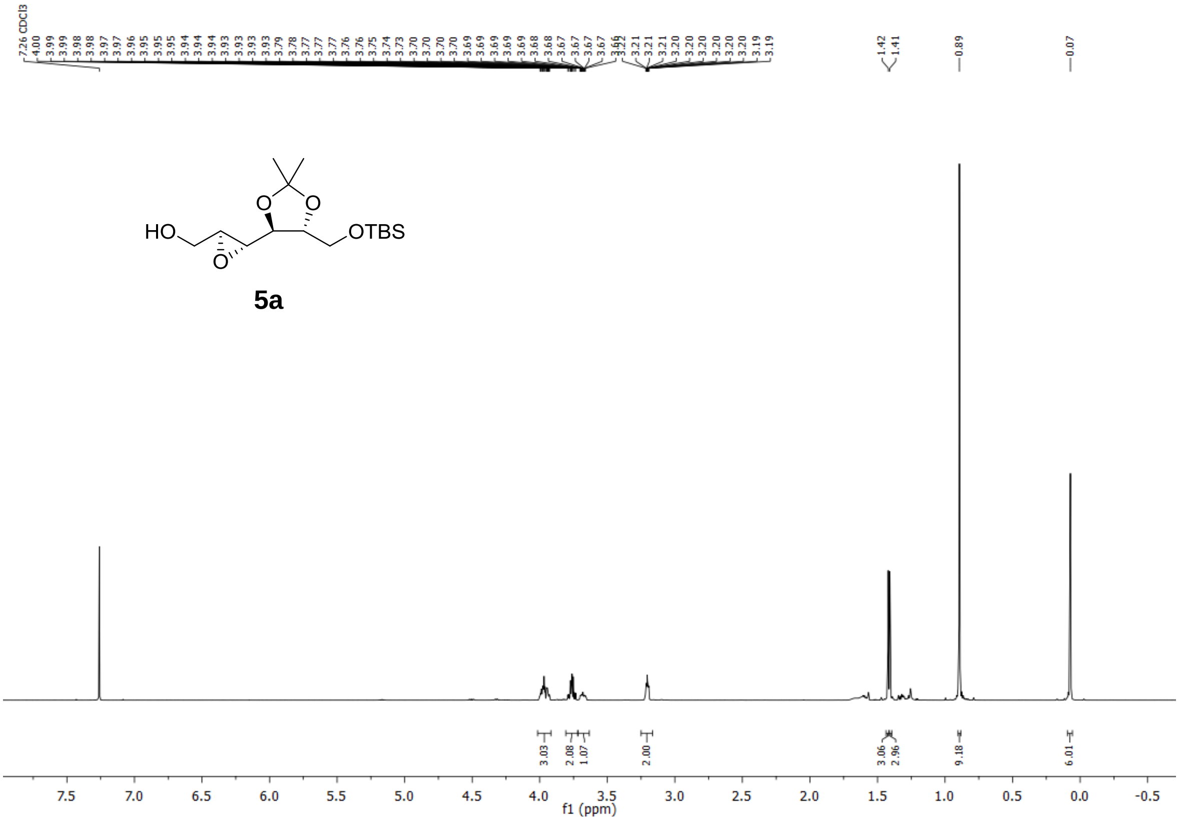

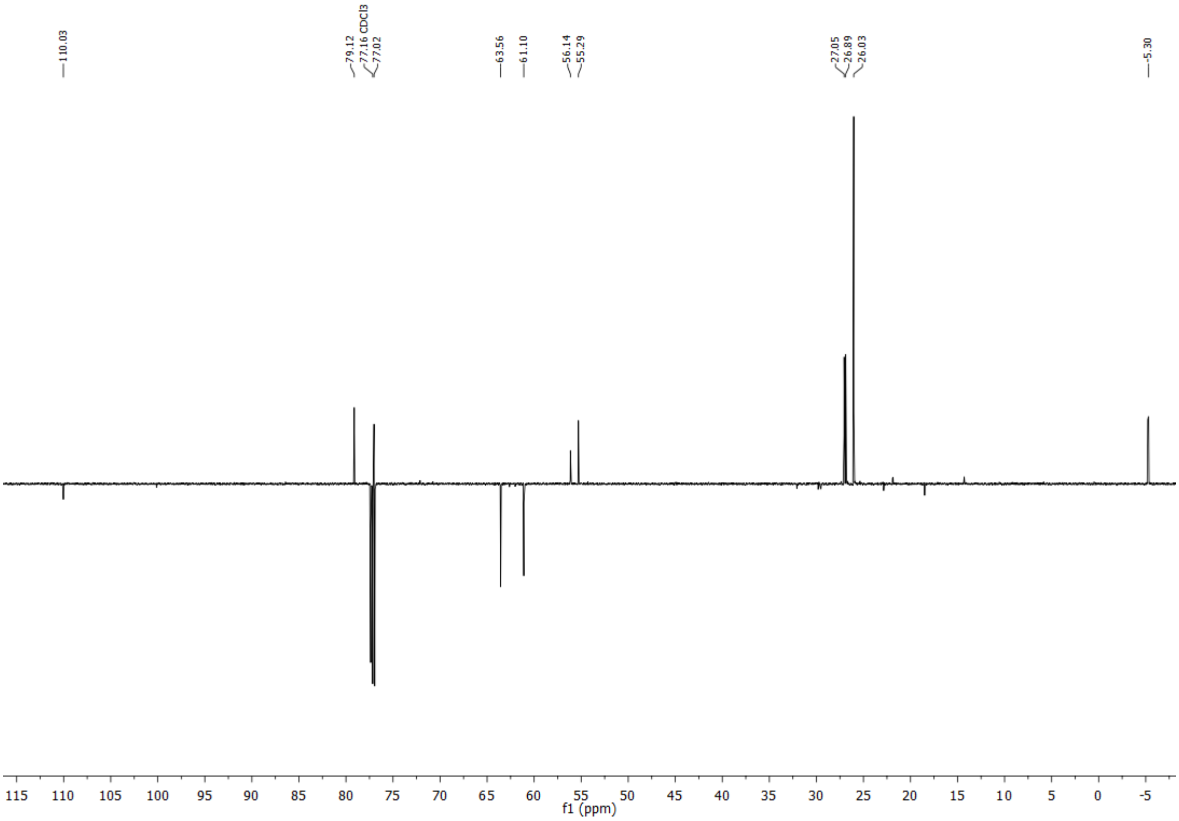

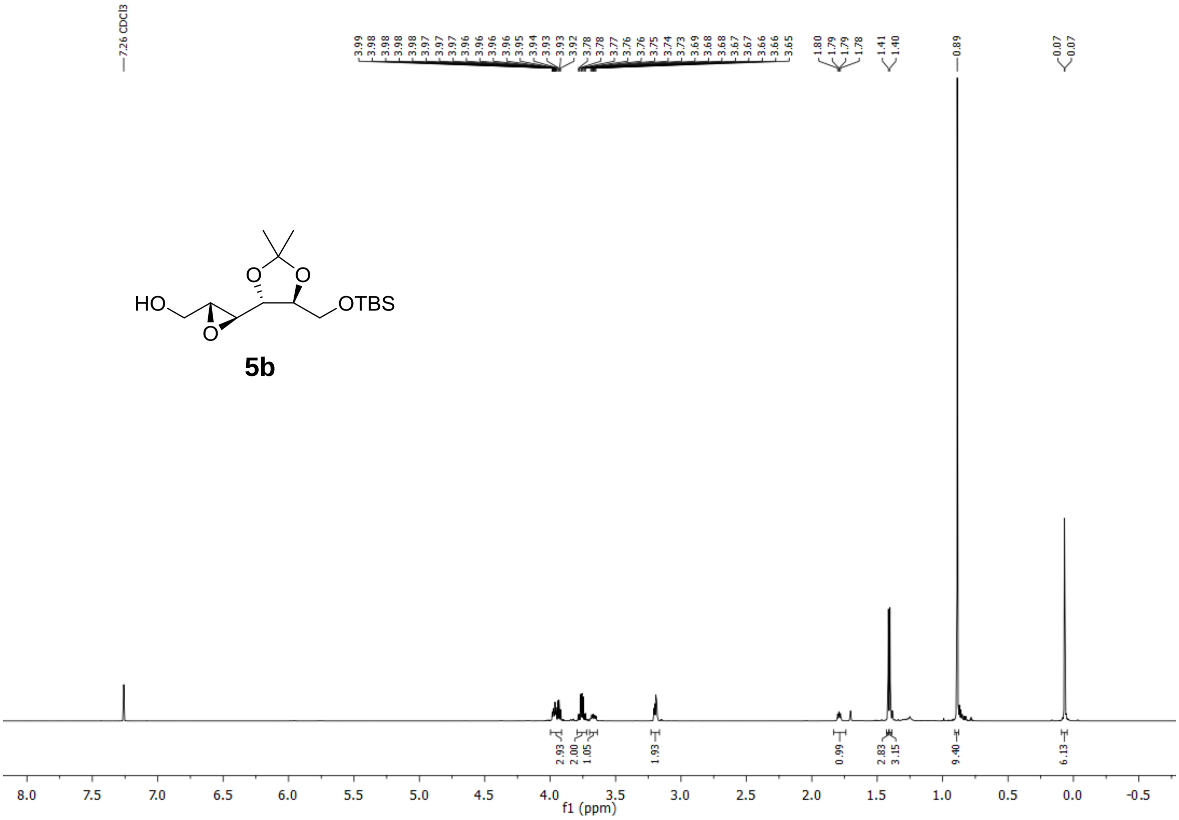
HO
O
O
O
OTBS
5b
7.26 CDCl3
f1 (ppm)

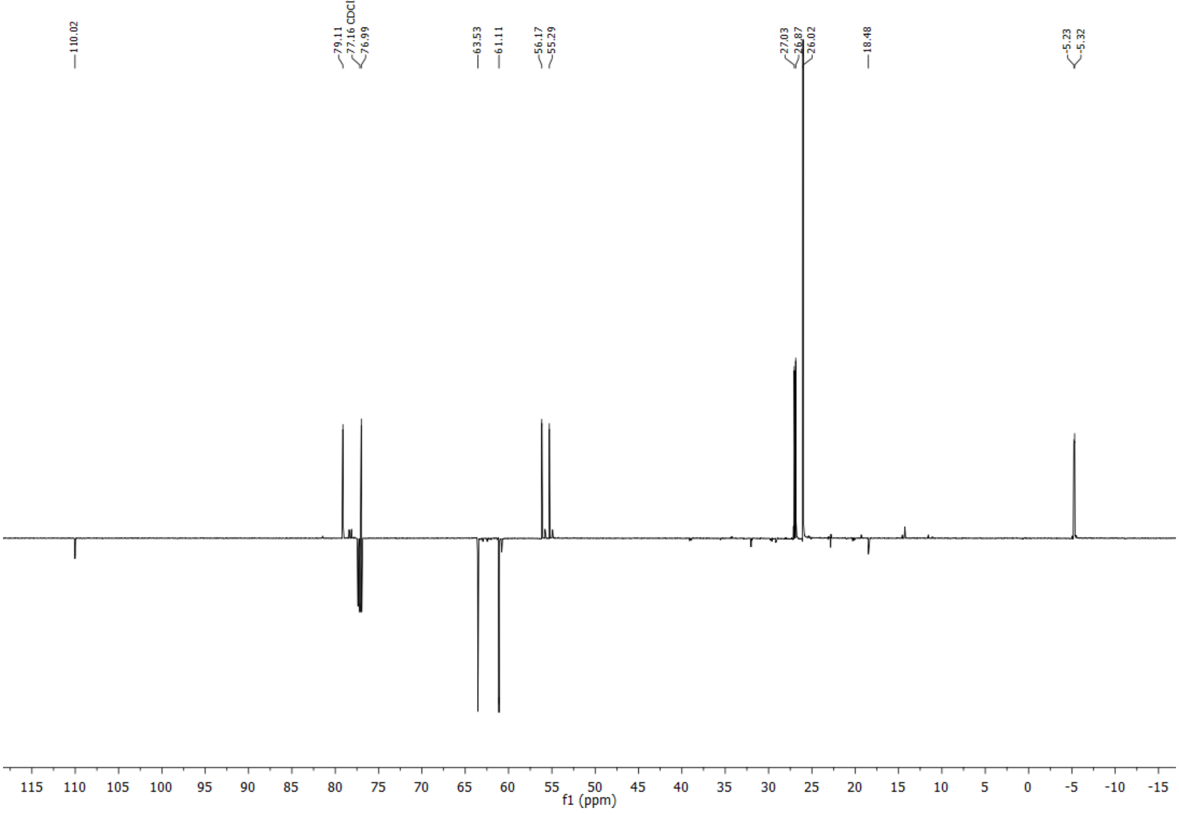
77.16 CDCl3
f1 (ppm)

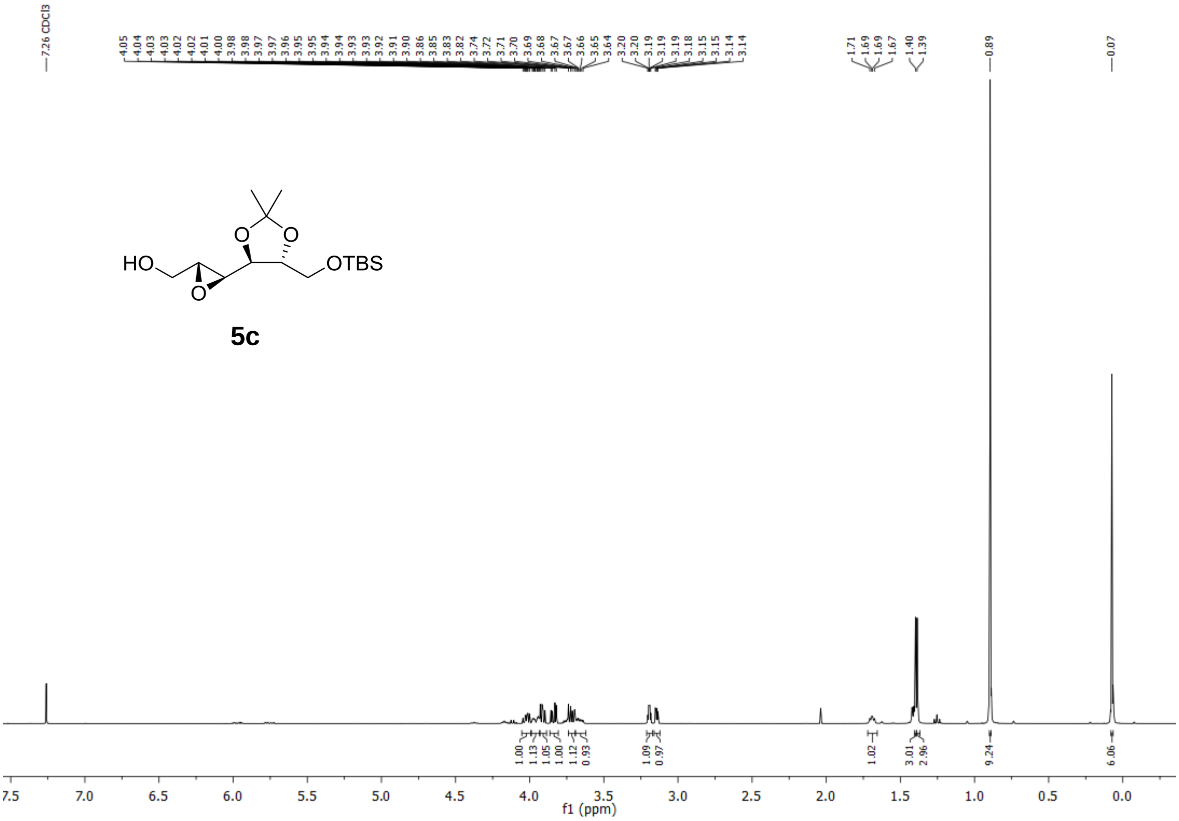

5c

7.26 CDCl3
4.05 4.04 4.03 4.03 4.02 4.02 4.01 4.00 3.98 3.98 3.97 3.97 3.96 3.95 3.95 3.94 3.94 3.93 3.93 3.92 3.91 3.90 3.86 3.85 3.83 3.82 3.74 3.72 3.71 3.70 3.69 3.68 3.67 3.67 3.66 3.65 3.64 3.20 3.19 3.19 3.19 3.18 3.15 3.15 3.14 3.14
1.71 1.69 1.69 1.67 1.40 1.39
0.89
0.07
1.00 1.13 1.05 1.00 1.12 0.93 1.09 0.97 1.02 3.01 2.96 9.24 6.06
f1 (ppm)

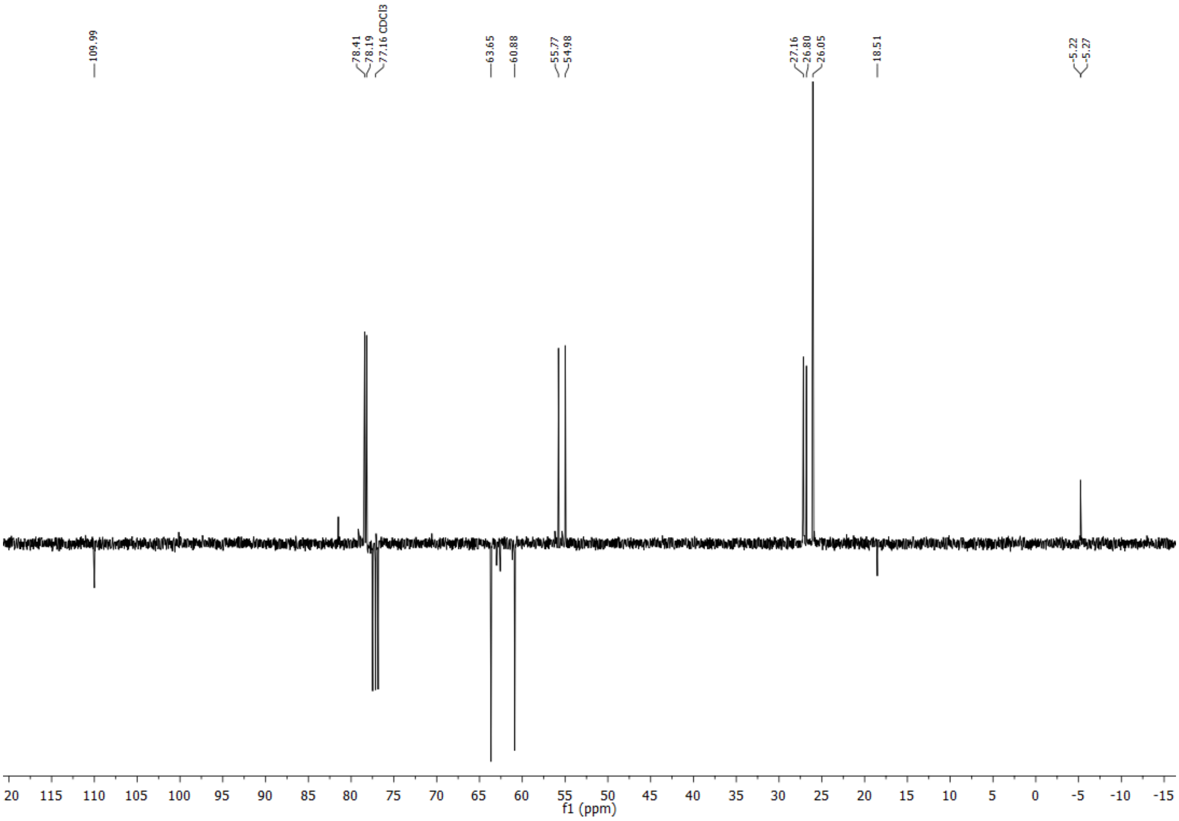
109.99
78.41 78.19 77.16 CDCl3
63.65
60.88
55.77 54.98
27.16 26.80 26.05
18.51
-5.22 -5.27
f1 (ppm)

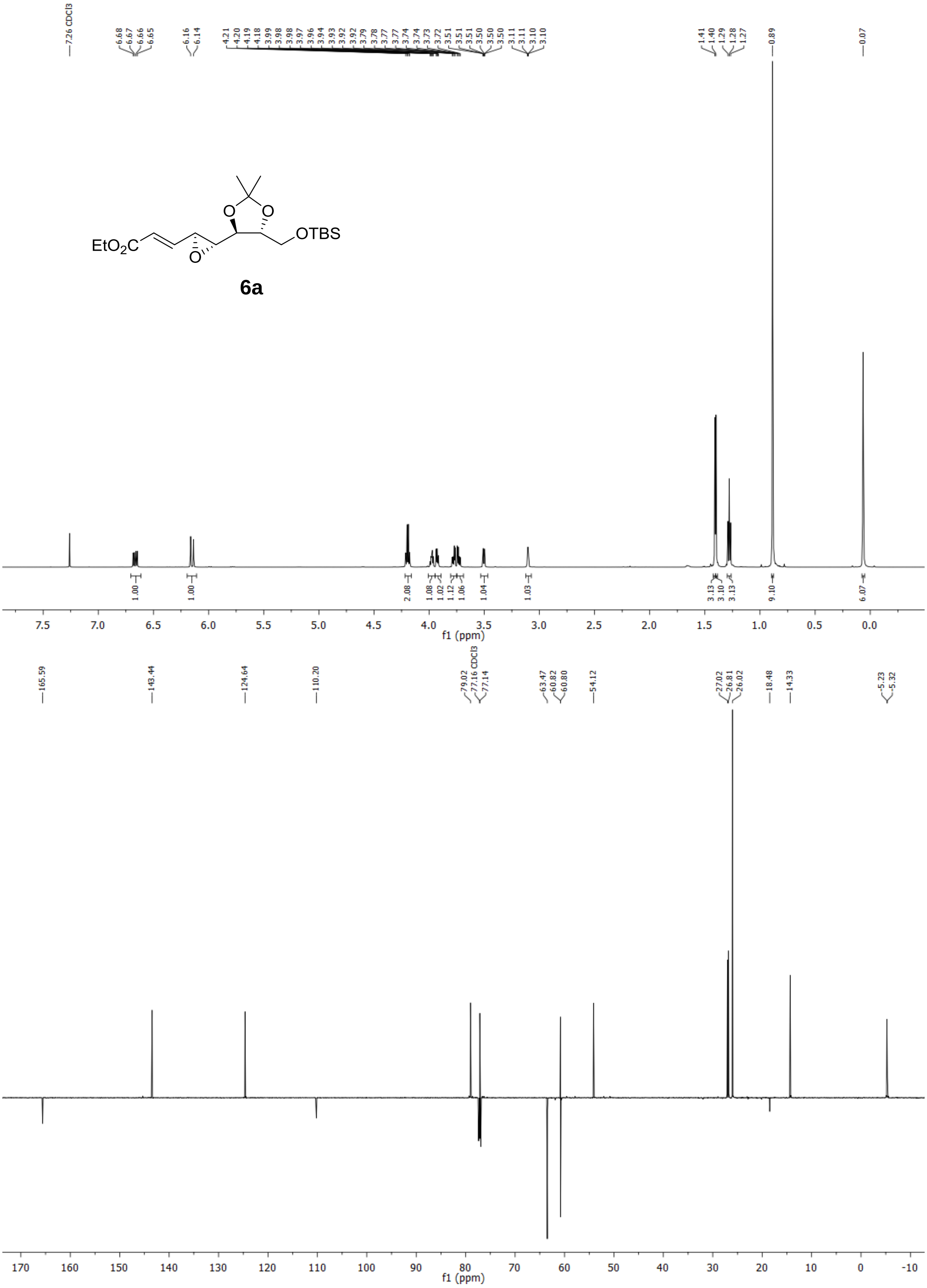

6a

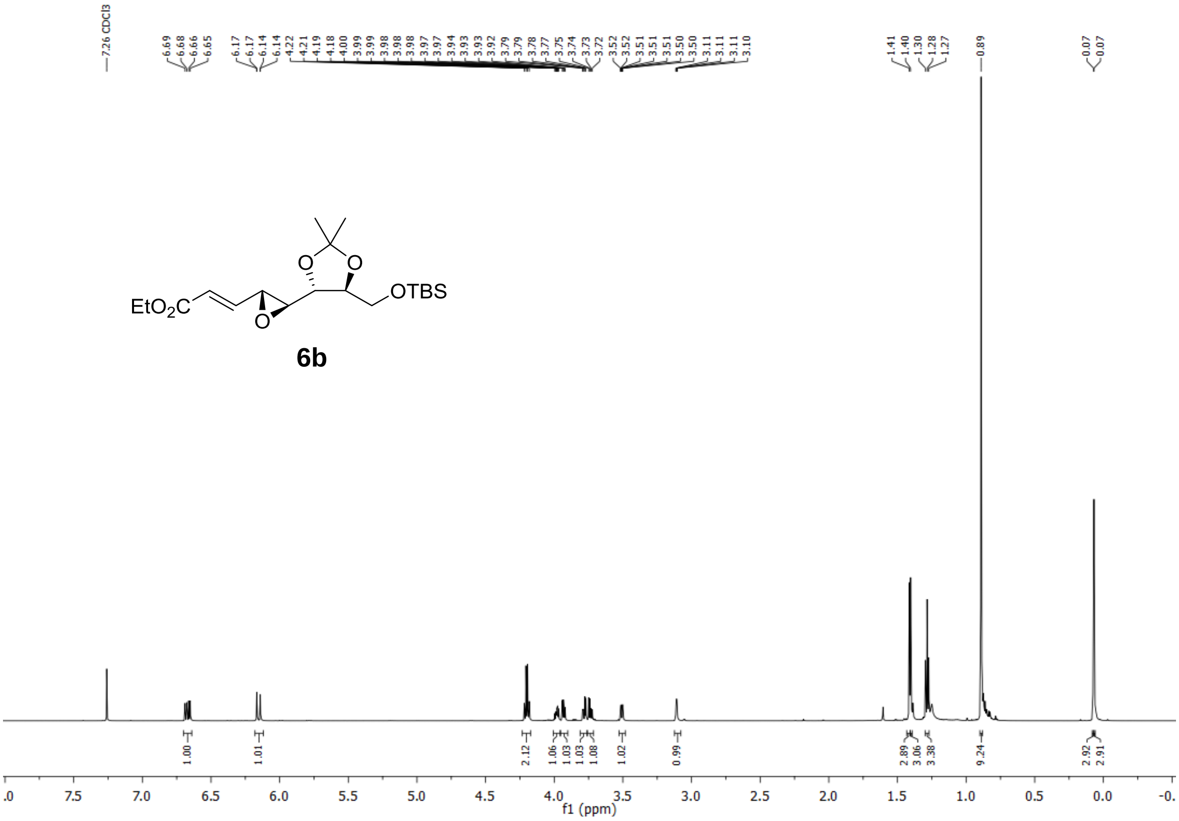
7.26 CDCl3
6.69 6.68 6.66 6.65
6.17 6.17 6.14 6.14
4.22 4.21 4.19 4.18 4.00 3.99 3.99 3.98 3.98 3.98 3.97 3.97 3.94 3.93 3.93 3.92 3.79 3.79 3.78 3.77 3.75 3.74 3.73 3.72 3.52 3.52 3.51 3.51 3.51 3.50 3.50 3.11 3.11 3.11 3.10
1.41 1.40 1.30 1.28 1.27
0.89
0.07 0.07
1.00 1.01 2.12 1.06 1.03 1.03 1.08 1.02 0.99 2.89 3.06 3.38 9.24 2.92 2.91
f1 (ppm)
EtO$_2$C
O
O
O
OTBS

6b

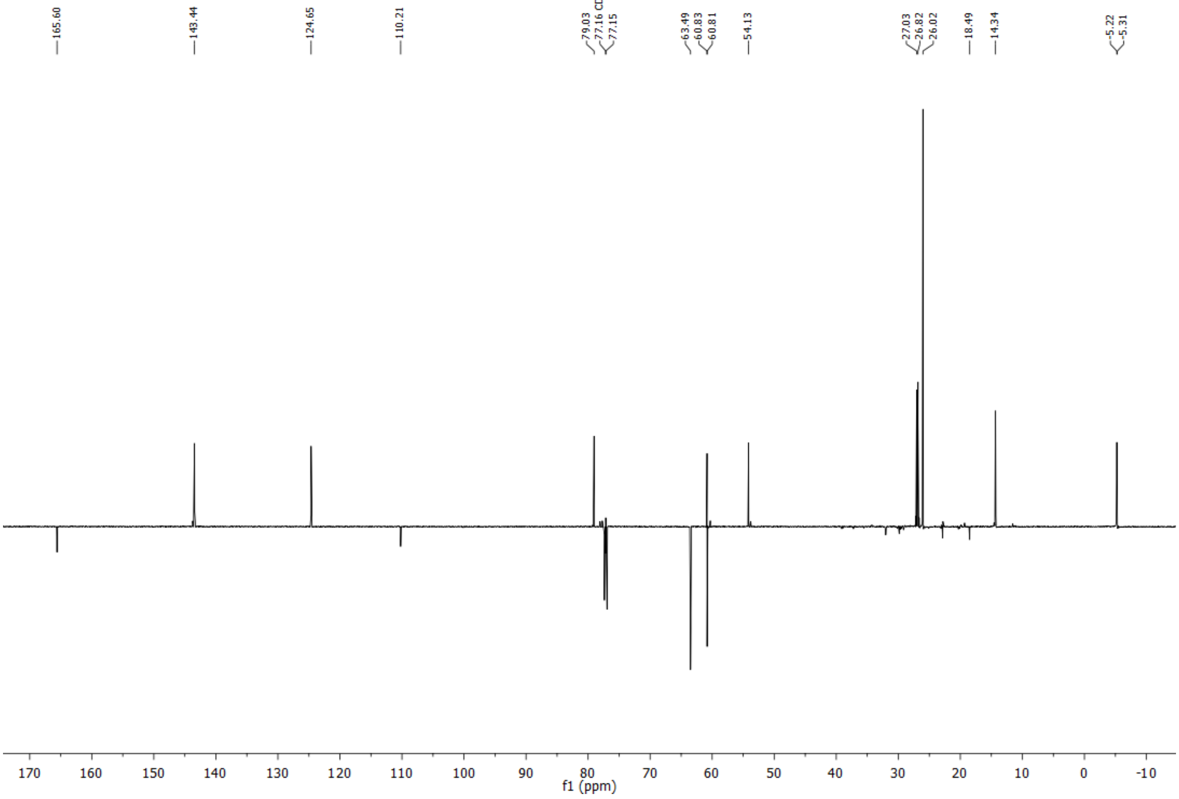
165.60
143.44
124.65
110.21
79.03 77.16 CDCl3 77.15
63.49 60.83 60.81
54.13
27.03 26.82 26.02
18.49
14.34
-5.22 -5.31
f1 (ppm)

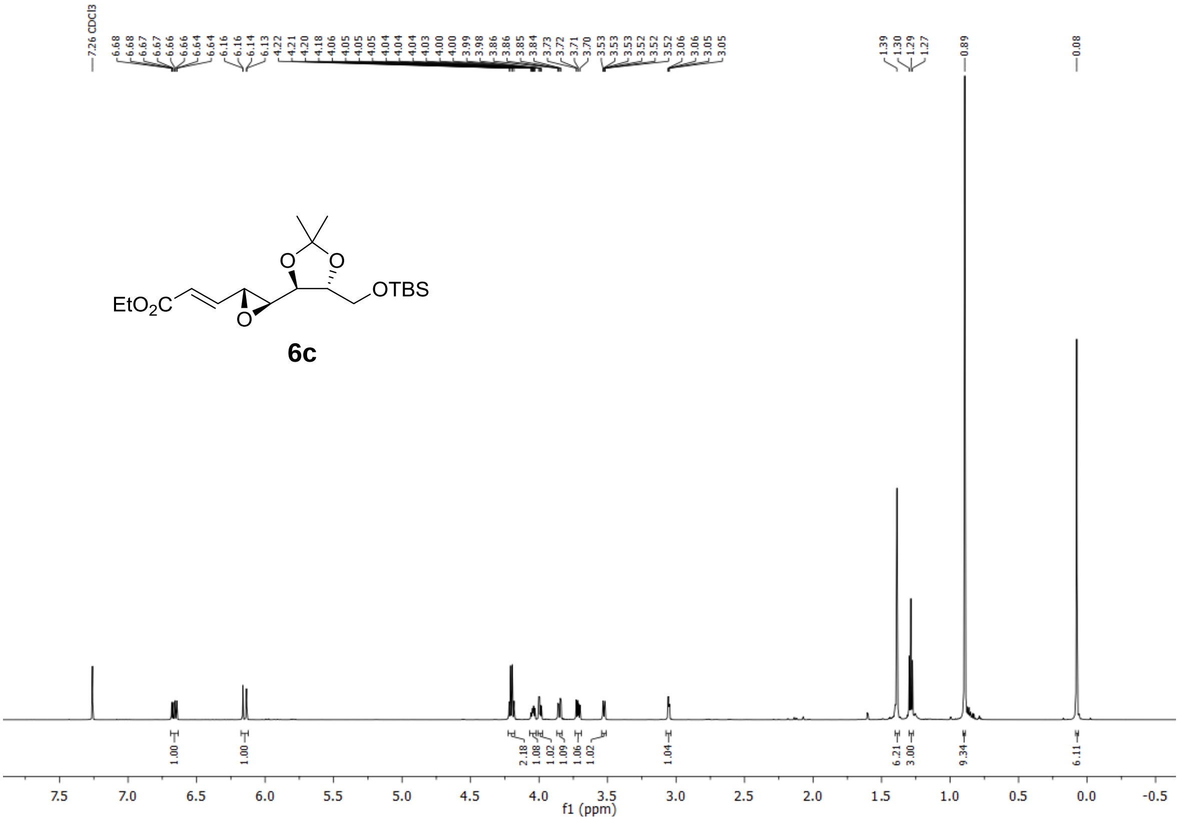
7.26 CDCl3
6.68 6.68 6.67 6.66 6.66 6.66 6.64 6.16 6.16 6.14 6.13 4.22 4.21 4.20 4.18 4.06 4.05 4.05 4.05 4.05 4.04 4.04 4.04 4.03 4.00 4.00 3.99 3.98 3.86 3.86 3.85 3.84 3.73 3.72 3.71 3.70 3.53 3.53 3.53 3.52 3.52 3.52 3.06 3.06 3.05 3.05
1.39 1.30 1.29 1.27
0.89
0.08
EtO2C
O
O
O
OTBS
6c
1.00 1.00 2.18 1.08 1.02 1.09 1.06 1.02 1.04 6.21 3.00 9.34 6.11
7.5 7.0 6.5 6.0 5.5 5.0 4.5 4.0 3.5 3.0 2.5 2.0 1.5 1.0 0.5 0.0 -0.5
f1 (ppm)

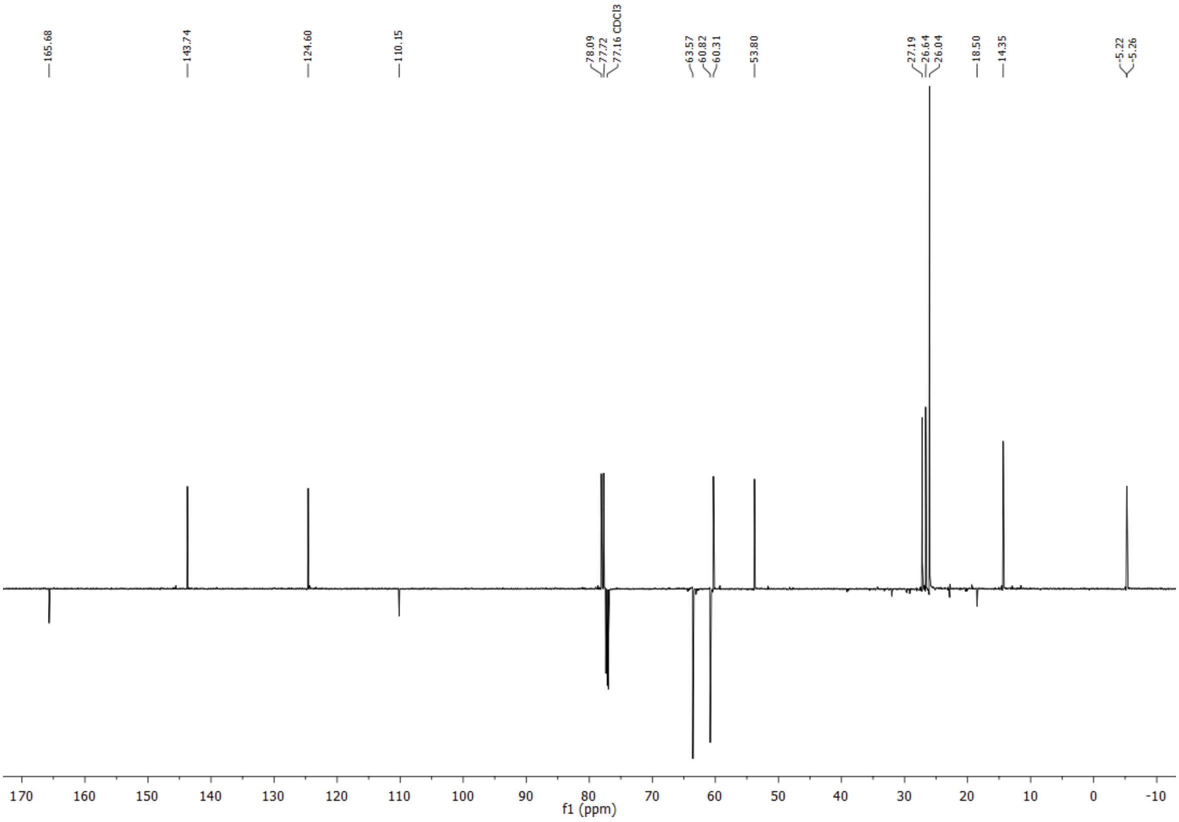
165.68
143.74
124.60
110.15
78.09 77.72 77.16 CDCl3
63.57 60.82 60.31
53.80
27.19 26.64 26.04
18.50
14.35
-5.22 -5.26
170 160 150 140 130 120 110 100 90 80 70 60 50 40 30 20 10 0 -10
f1 (ppm)

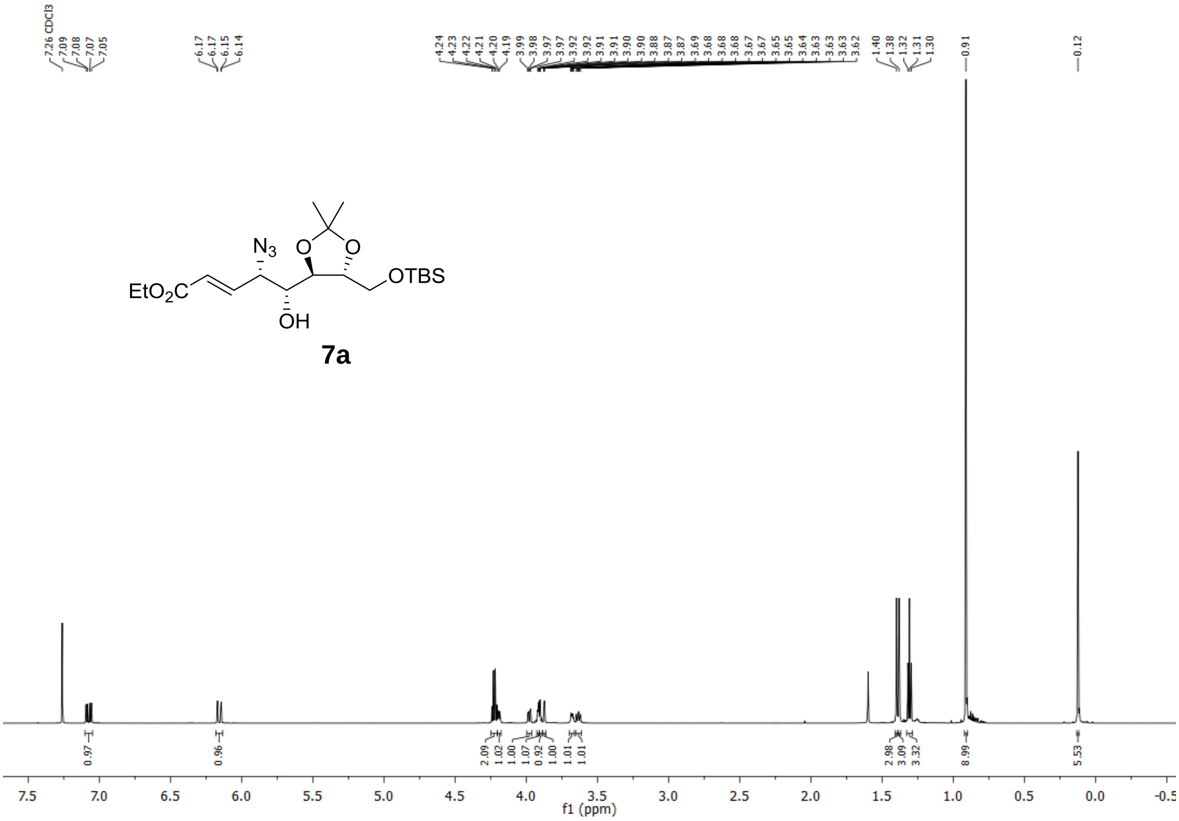
7.26 CDCl3
7.09
7.08
7.07
7.05
6.17
6.17
6.15
6.14
4.24
4.23
4.22
4.21
4.20
4.19
3.99
3.98
3.97
3.97
3.92
3.92
3.91
3.91
3.90
3.90
3.88
3.87
3.87
3.69
3.68
3.68
3.68
3.67
3.67
3.65
3.65
3.64
3.63
3.63
3.63
3.62
1.40
1.38
1.32
1.31
1.30
0.91
0.12
N3
O
O
EtO2C
OTBS
OH
7a
0.97
0.96
2.09
1.02
1.00
1.07
0.92
1.00
1.01
1.01
2.98
3.09
3.32
8.99
5.53
f1 (ppm)

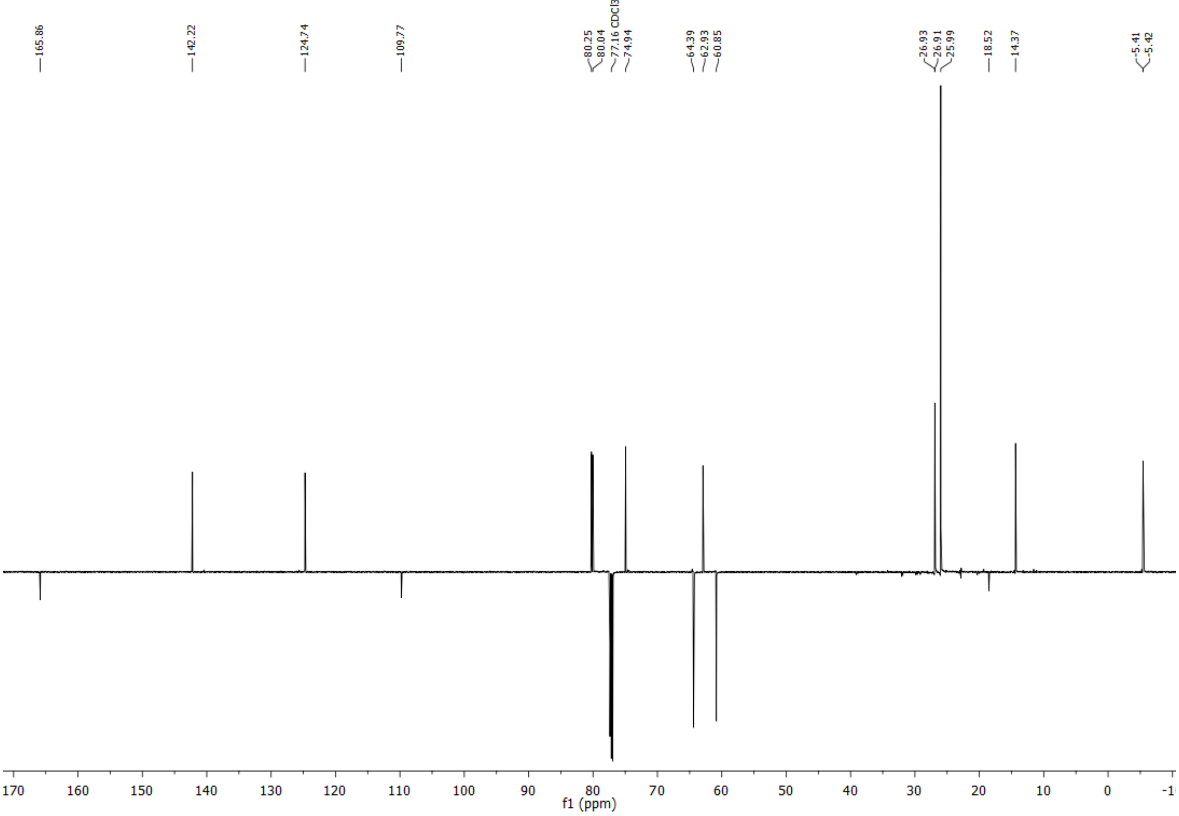
165.86
142.22
124.74
109.77
80.25
80.04
77.16 CDCl3
74.94
64.39
62.93
60.85
26.93
26.91
25.99
18.52
14.37
-5.41
-5.42
f1 (ppm)

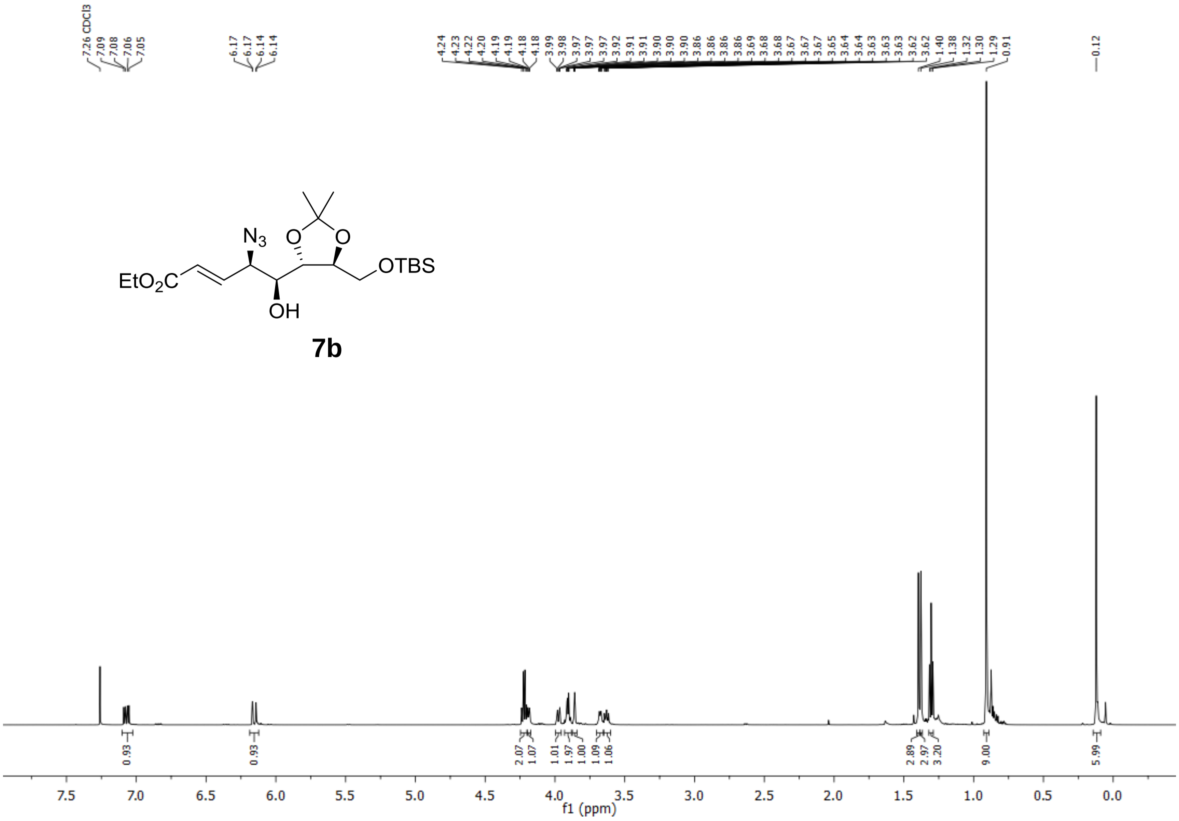

7b

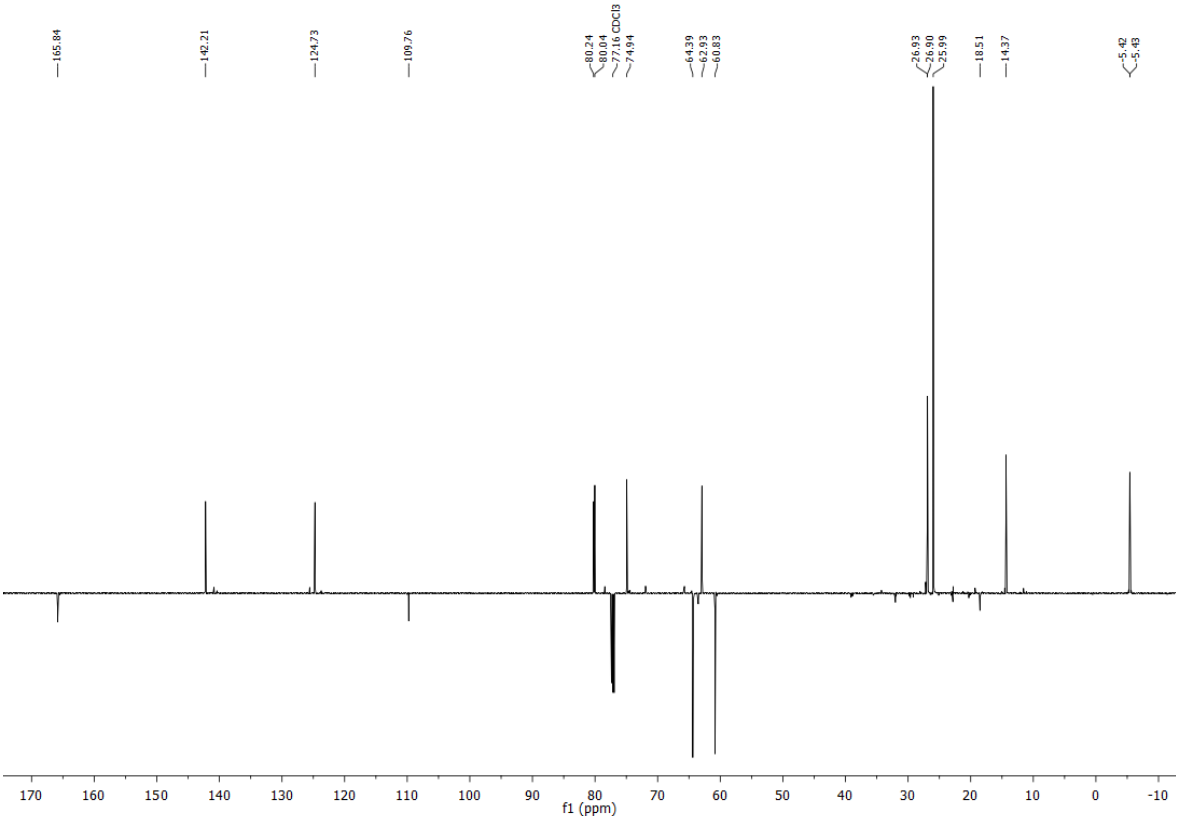

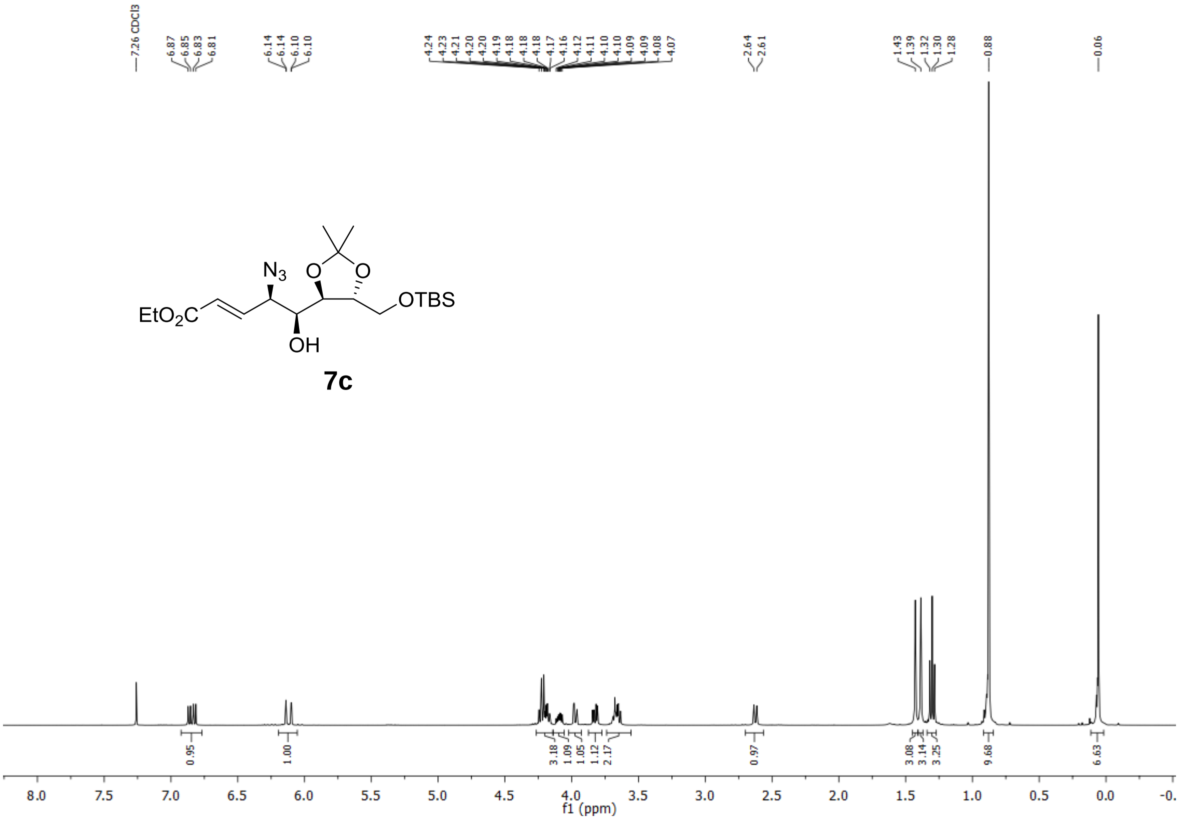
N3
O
O
OTBS
EtO2C
OH
7c
f1 (ppm)

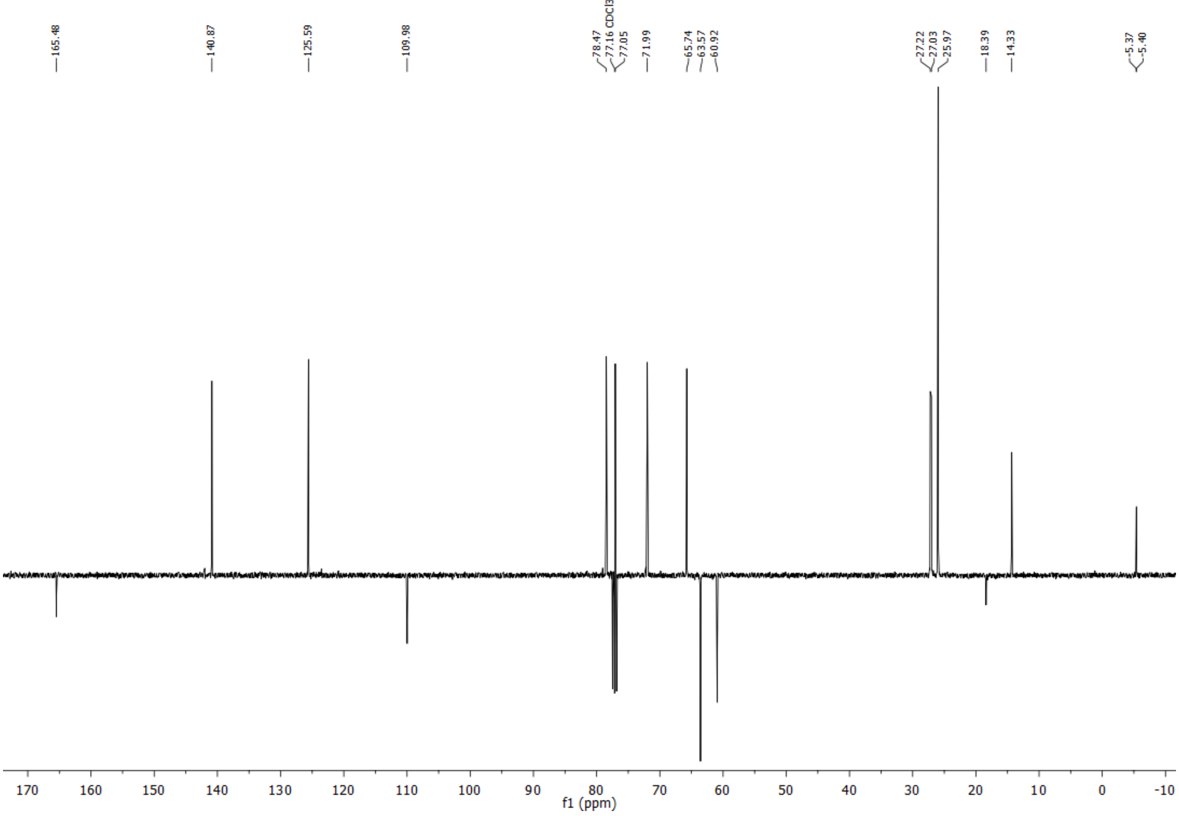
f1 (ppm)

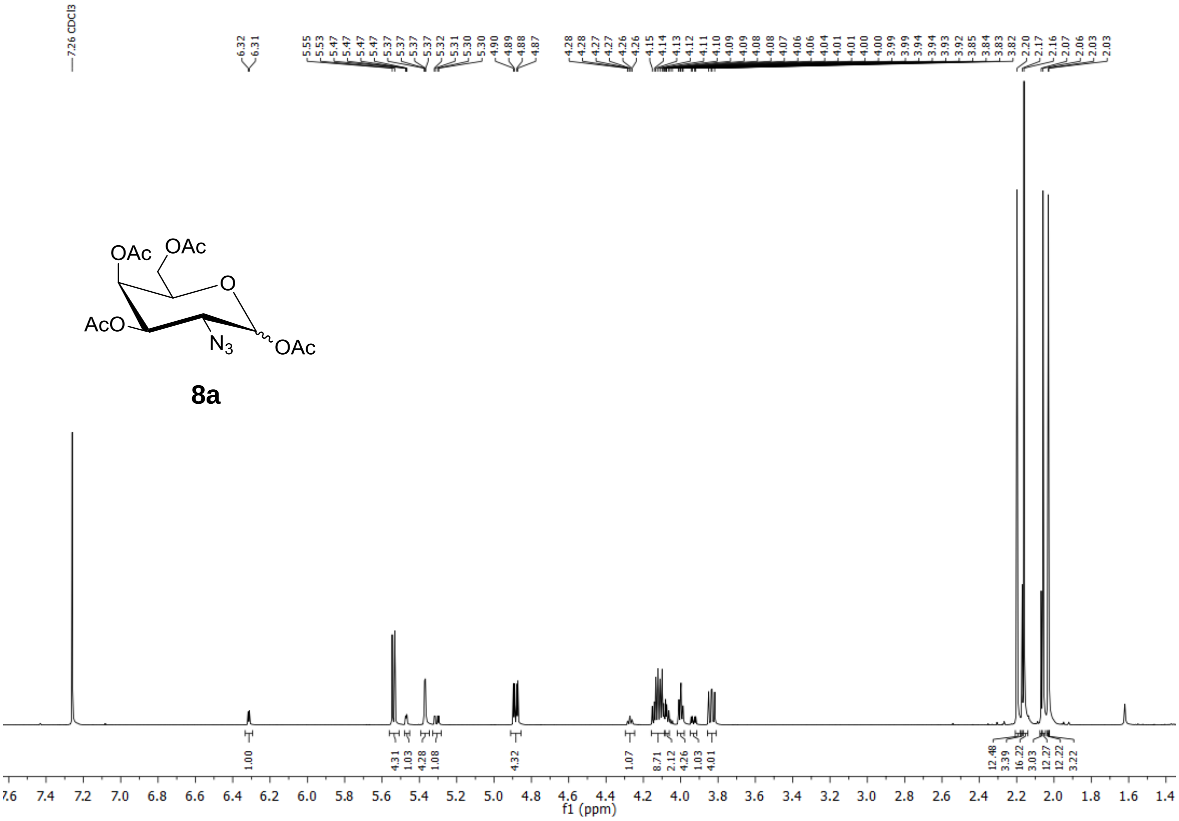
7.26 CDCl3
6.32
6.31
5.55 5.53 5.47 5.47 5.47 5.47 5.37 5.37 5.37 5.37 5.32 5.31 5.30 5.30 4.90 4.89 4.88 4.87
4.28 4.28 4.27 4.27 4.26 4.26 4.15 4.14 4.13 4.12 4.11 4.10 4.09 4.09 4.08 4.08 4.07 4.06 4.06 4.04 4.04 4.01 4.01 4.00 4.00 3.99 3.99 3.94 3.94 3.93 3.92 3.85 3.84 3.83 3.82 2.20 2.17 2.16 2.07 2.06 2.03 2.03
1.00 4.31 1.03 4.28 1.08 4.32 1.07 8.71 2.12 4.26 1.03 4.01 12.48 3.39 16.22 3.03 12.27 12.22 3.22
f1 (ppm)
OAc
OAc
O
AcO
N3
OAc

8a

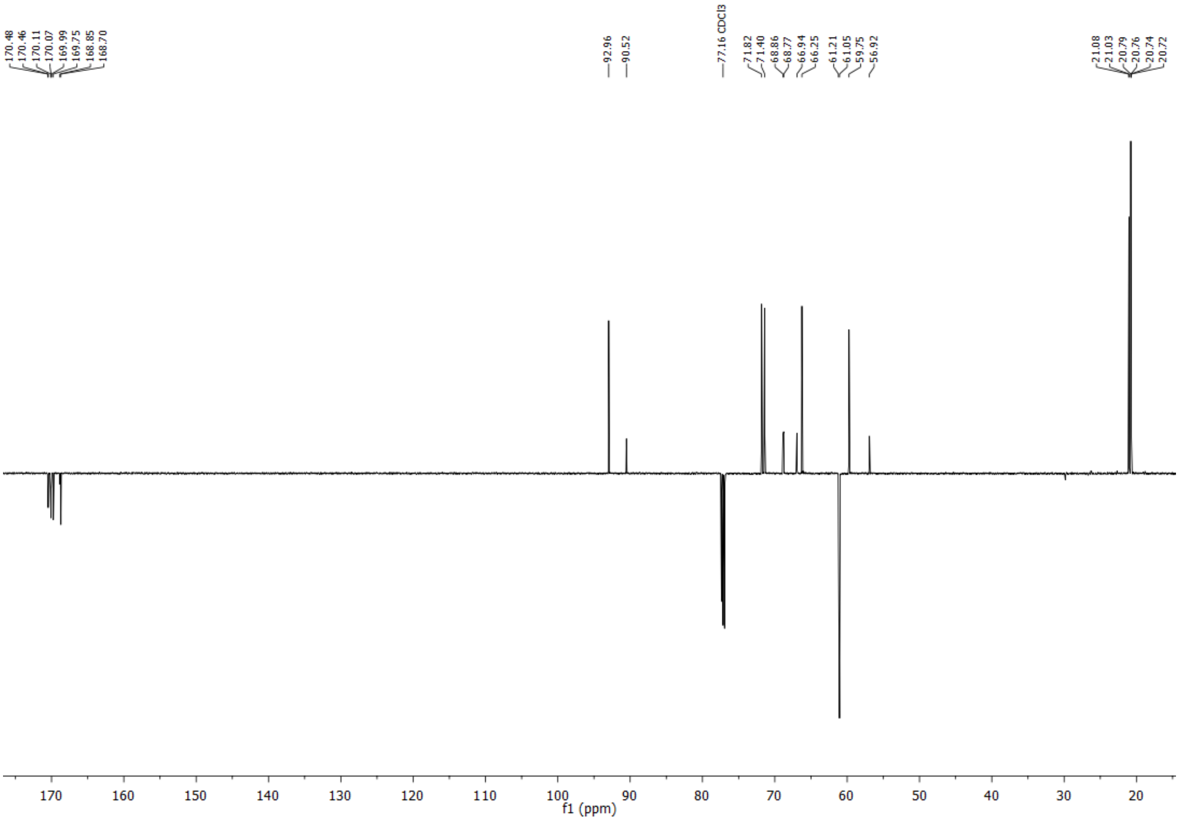
170.48 170.46 170.11 170.07 169.99 169.75 168.85 168.70
92.96
90.52
77.16 CDCl3
71.82 71.40 68.86 68.77 66.94 66.25 61.21 61.05 59.75 56.92
21.08 21.03 20.79 20.76 20.74 20.72
f1 (ppm)

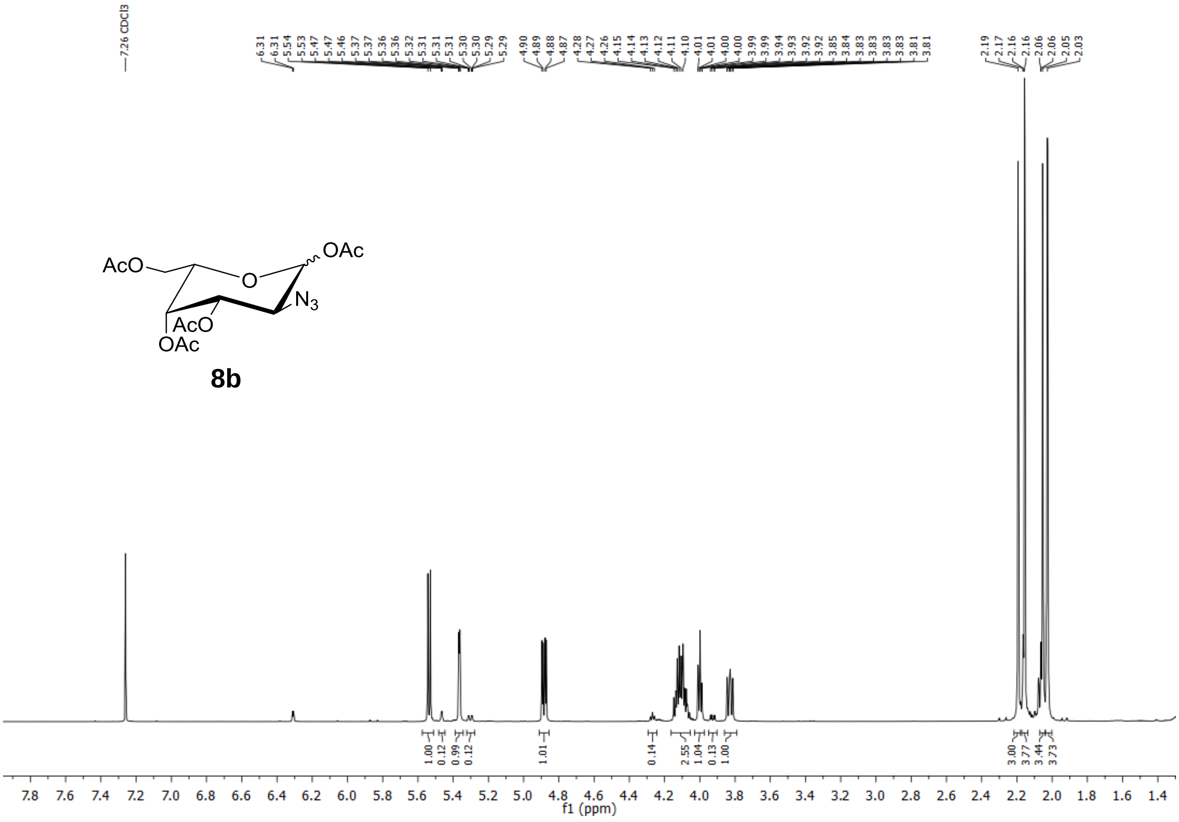

7.26 CDCl3
6.31 6.31 5.54 5.53 5.47 5.47 5.46 5.37 5.37 5.37 5.36 5.36 5.36 5.32 5.31 5.31 5.31 5.30 5.30 5.29 5.29
4.90 4.89 4.88 4.87 4.28 4.27 4.26 4.15 4.14 4.13 4.12 4.11 4.10 4.01 4.01 4.00 4.00 3.99 3.99 3.94 3.93 3.93 3.92 3.92 3.85 3.84 3.83 3.83 3.83 3.83 3.81 3.81
2.19 2.17 2.16 2.16 2.06 2.06 2.05 2.03
1.00 0.12 0.99 0.12 1.01 0.14 2.55 1.04 0.13 1.00 3.00 3.77 3.44 3.73
f1 (ppm)
OAc
AcO
O
N3
AcO
OAc

8b

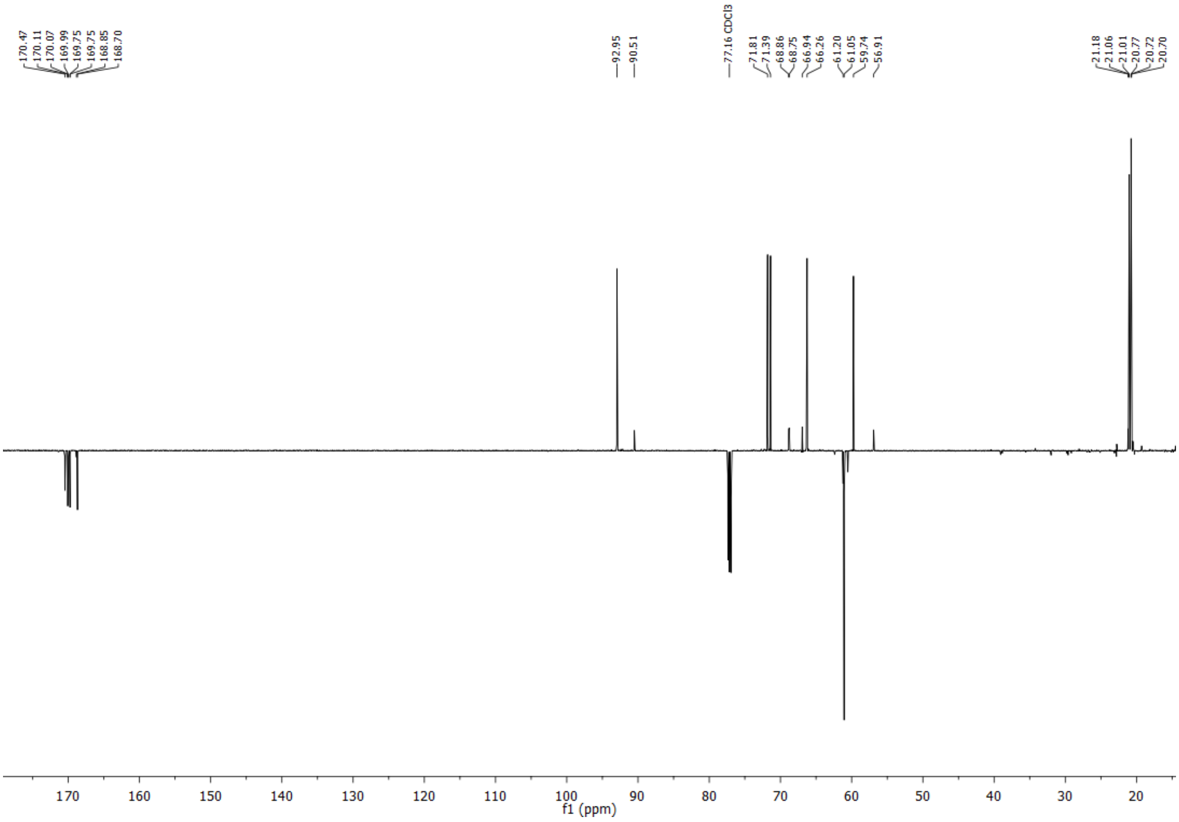

170.47 170.11 170.07 169.99 169.75 169.75 168.85 168.70
92.95 90.51
77.16 CDCl3
71.81 71.39 68.86 68.75 66.94 66.26 61.20 61.05 59.74 56.91
21.18 21.06 21.01 20.77 20.72 20.70
f1 (ppm)

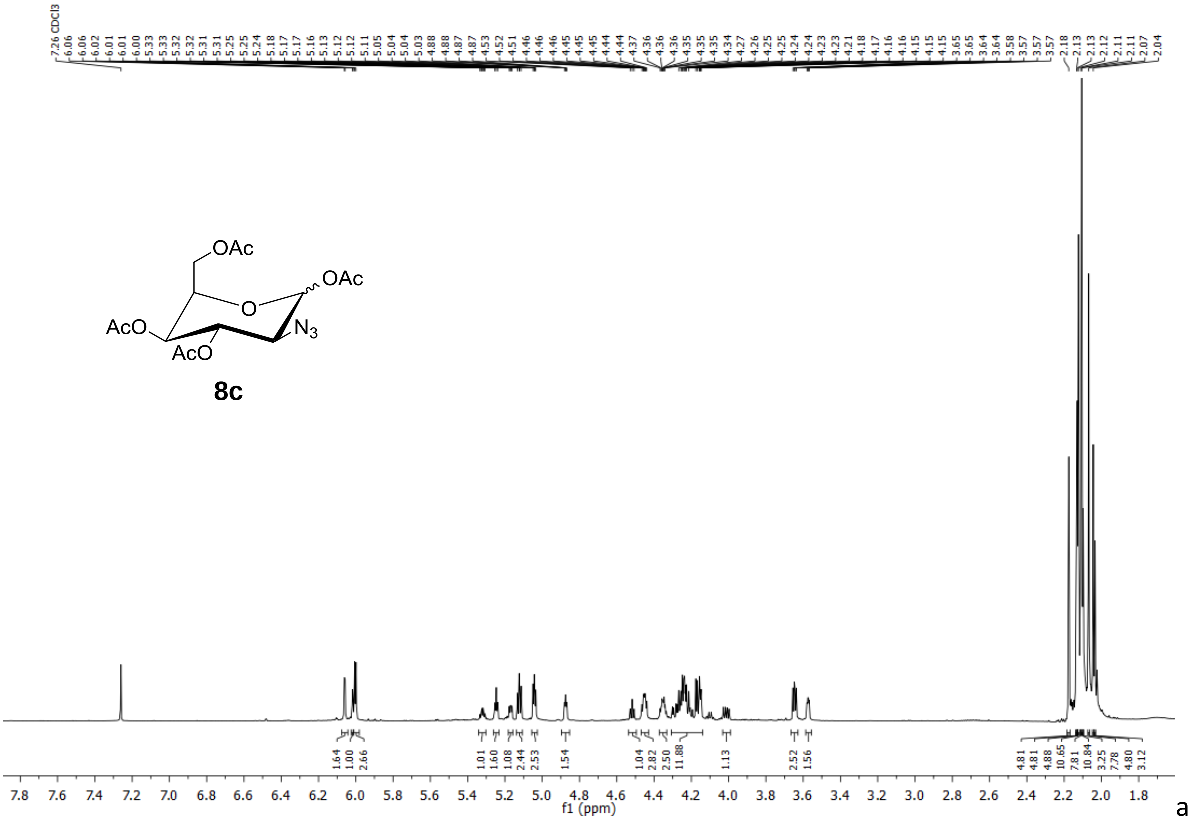
OAc
OAc
O
AcO
AcO
N3

8c

7.26 CDCl3
6.06
6.06
6.02
6.01
6.01
6.00
5.33
5.33
5.32
5.32
5.31
5.31
5.25
5.25
5.24
5.18
5.17
5.17
5.16
5.13
5.12
5.12
5.11
5.05
5.04
5.04
5.03
4.88
4.88
4.87
4.87
4.53
4.52
4.51
4.46
4.46
4.46
4.45
4.45
4.45
4.44
4.37
4.36
4.36
4.36
4.35
4.35
4.35
4.34
4.27
4.26
4.25
4.25
4.24
4.24
4.23
4.21
4.18
4.17
4.16
4.16
4.15
4.15
4.15
3.65
3.65
3.64
3.64
3.58
3.57
3.57
2.18
2.13
2.13
2.12
2.11
2.11
2.07
2.04
1.64
1.00
2.66
1.01
1.60
1.08
2.44
2.53
1.54
1.04
2.82
2.50
11.88
1.13
2.52
1.56
4.81
4.81
4.88
10.65
7.81
10.84
3.25
7.78
4.80
3.12
7.8 7.6 7.4 7.2 7.0 6.8 6.6 6.4 6.2 6.0 5.8 5.6 5.4 5.2 5.0 4.8 4.6 4.4 4.2 4.0 3.8 3.6 3.4 3.2 3.0 2.8 2.6 2.4 2.2 2.0 1.8
f1 (ppm)

a

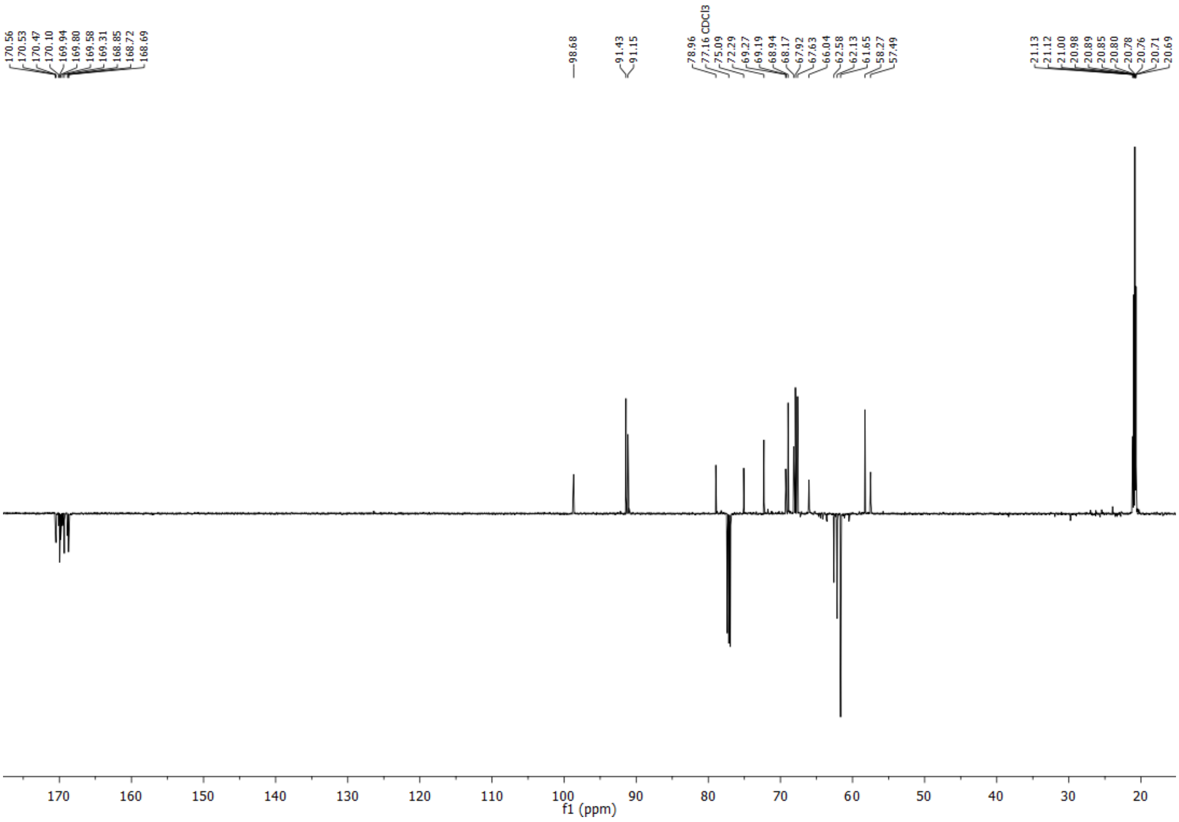
170.56
170.53
170.47
170.10
169.94
169.80
169.58
169.31
168.85
168.72
168.69
98.68
91.43
91.15
78.96
77.16 CDCl3
75.09
72.29
69.27
69.19
68.94
68.17
67.92
67.63
66.04
62.58
62.13
61.65
58.27
57.49
21.13
21.12
21.00
20.98
20.89
20.85
20.80
20.78
20.76
20.71
20.69
170 160 150 140 130 120 110 100 90 80 70 60 50 40 30 20
f1 (ppm)

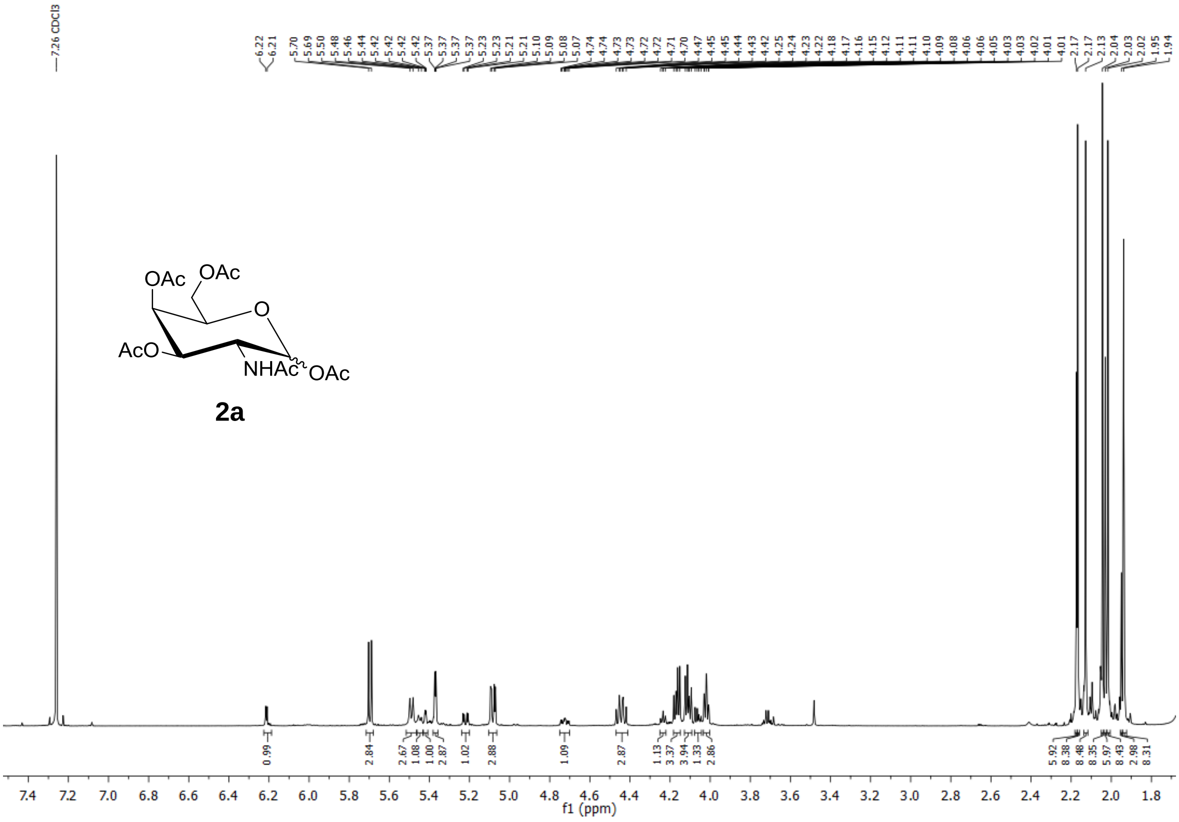

7.26 CDCl3
6.22
6.21
5.70
5.69
5.50
5.48
5.46
5.44
5.42
5.42
5.42
5.37
5.37
5.37
5.37
5.23
5.23
5.21
5.10
5.09
5.08
5.07
4.74
4.74
4.73
4.73
4.72
4.72
4.71
4.70
4.47
4.45
4.45
4.44
4.43
4.42
4.25
4.24
4.23
4.22
4.18
4.17
4.16
4.15
4.12
4.11
4.11
4.10
4.09
4.08
4.06
4.05
4.03
4.03
4.02
4.01
4.01
2.17
2.17
2.13
2.04
2.03
2.02
1.95
1.94
OAc
OAc
O
AcO
NHAc
OAc
2a
0.99
2.84
2.67
1.08
1.00
2.87
1.02
2.88
1.09
2.87
1.13
3.37
3.94
1.33
2.86
5.92
8.38
8.48
8.35
5.97
8.43
2.98
8.31
7.4 7.2 7.0 6.8 6.6 6.4 6.2 6.0 5.8 5.6 5.4 5.2 5.0 4.8 4.6 4.4 4.2 4.0 3.8 3.6 3.4 3.2 3.0 2.8 2.6 2.4 2.2 2.0 1.8
f1 (ppm)

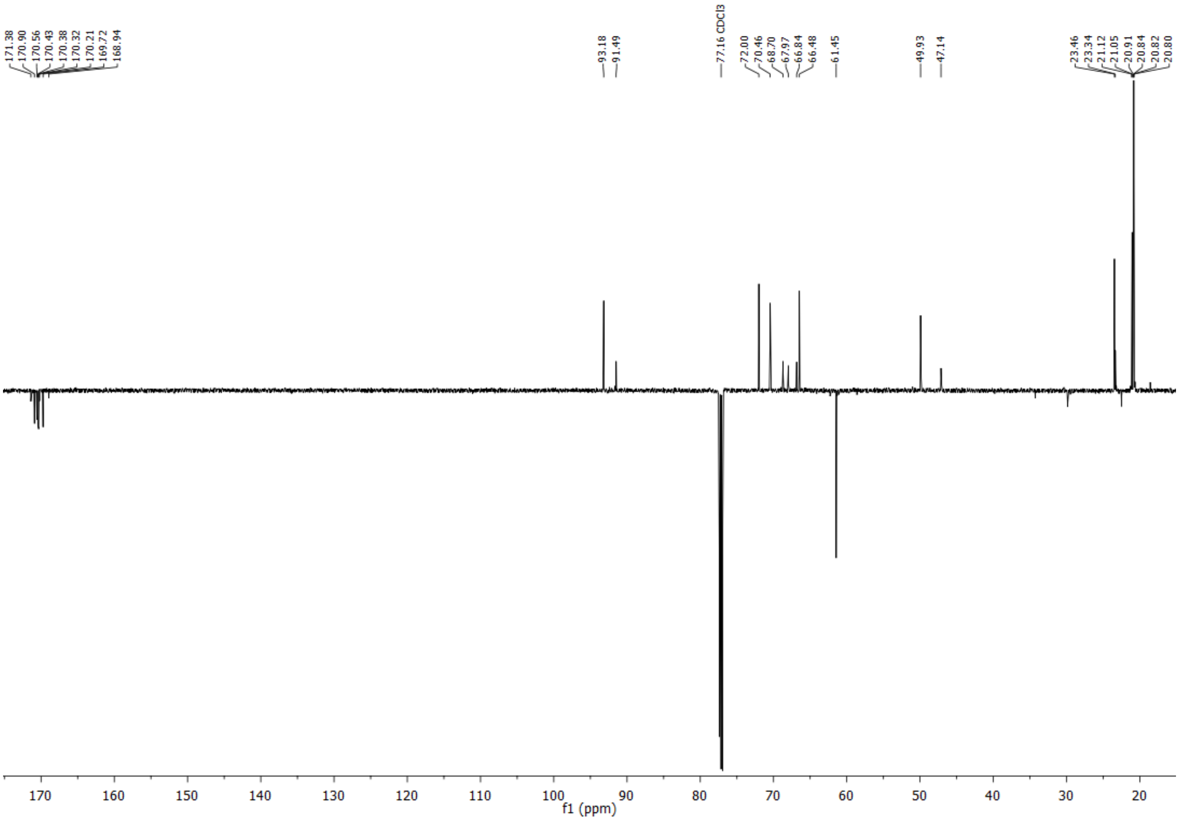

171.38
170.90
170.56
170.43
170.38
170.32
170.21
169.72
168.94
93.18
91.49
77.16 CDCl3
72.00
70.46
68.70
67.97
66.84
66.48
61.45
49.93
47.14
23.46
23.34
21.12
21.05
20.91
20.84
20.82
20.80
170 160 150 140 130 120 110 100 90 80 70 60 50 40 30 20
f1 (ppm)

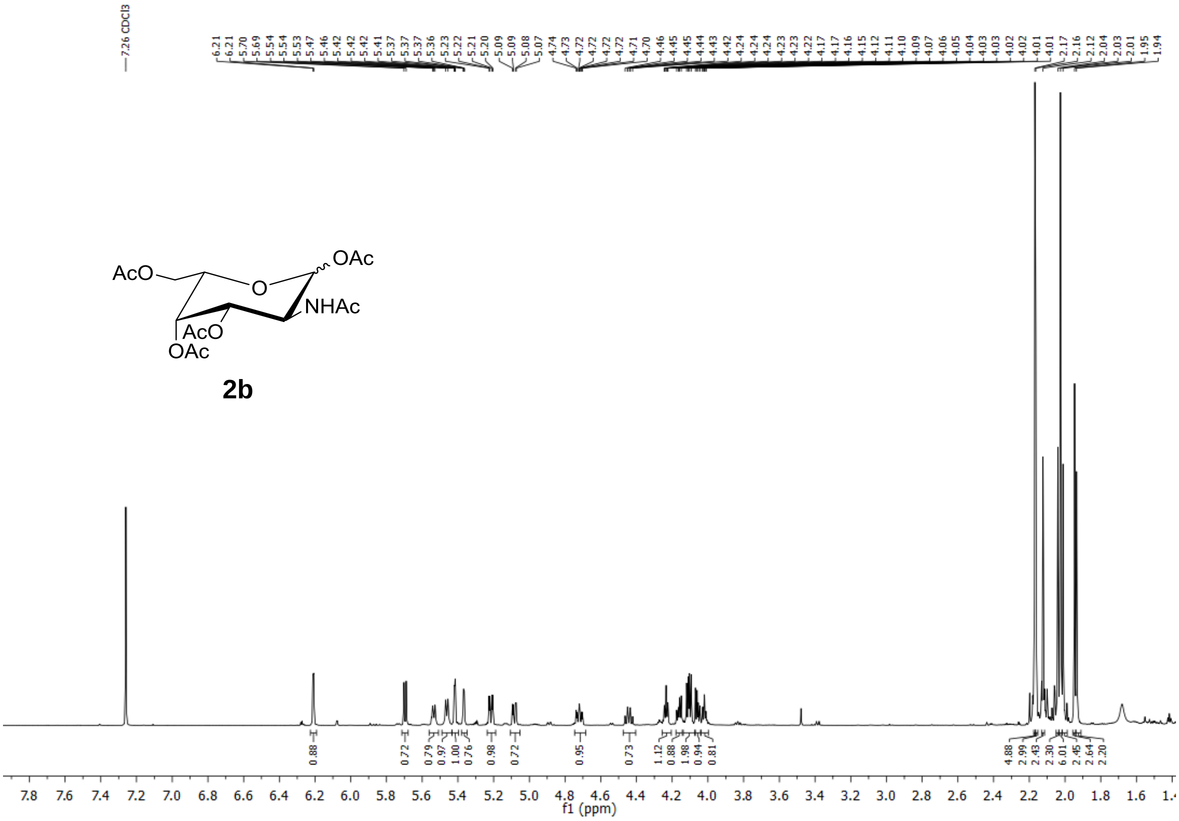

2b

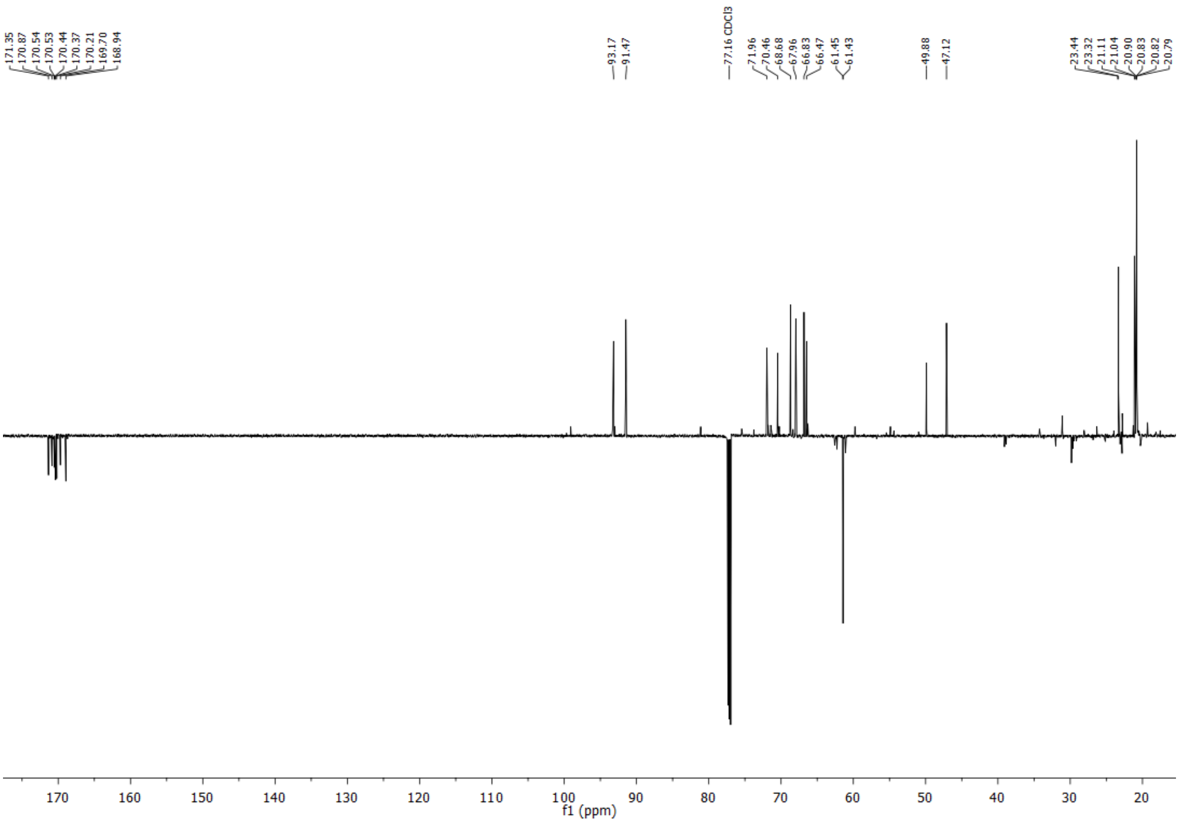

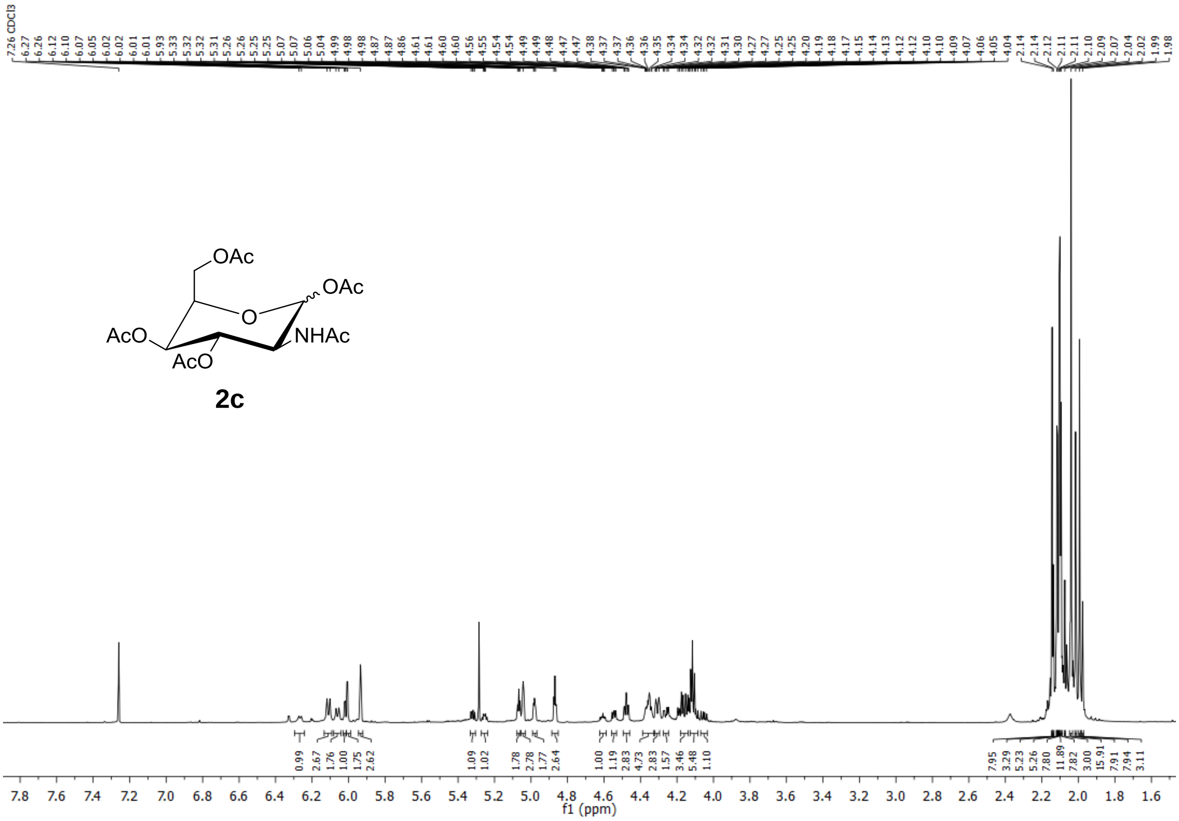

2c

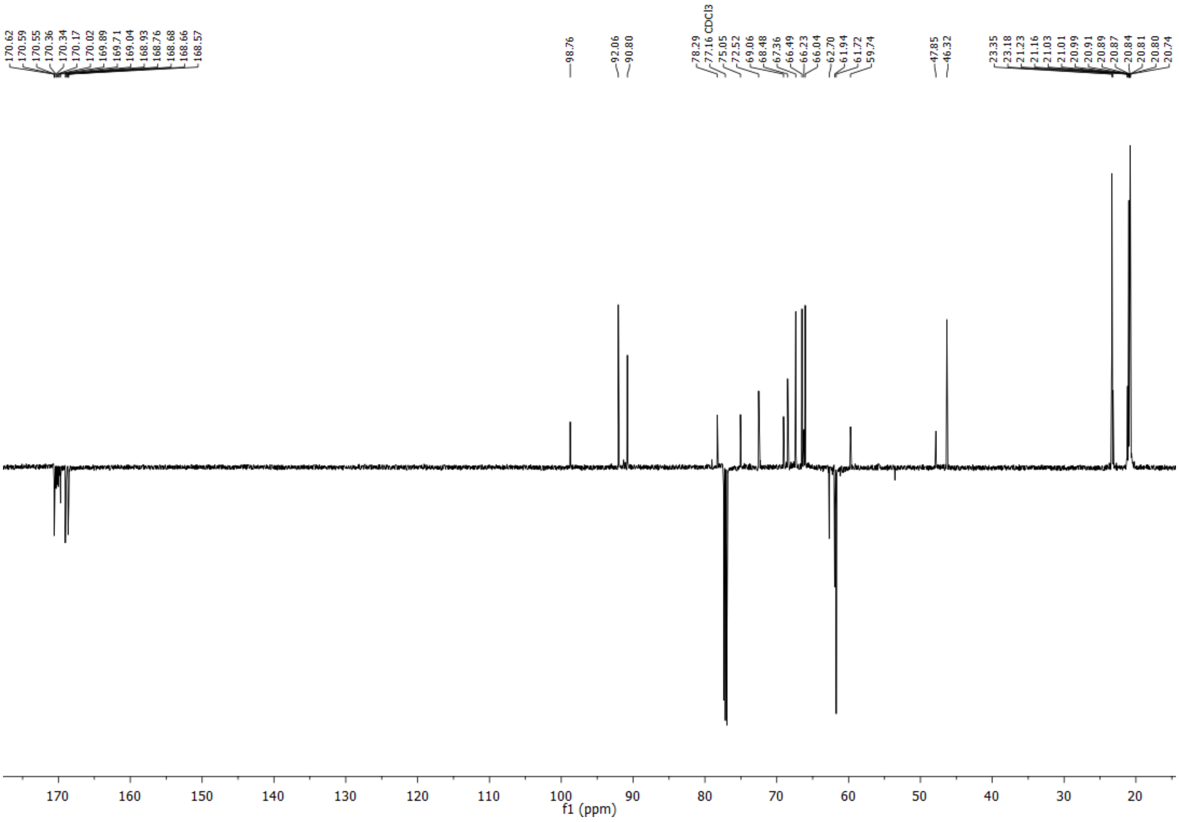

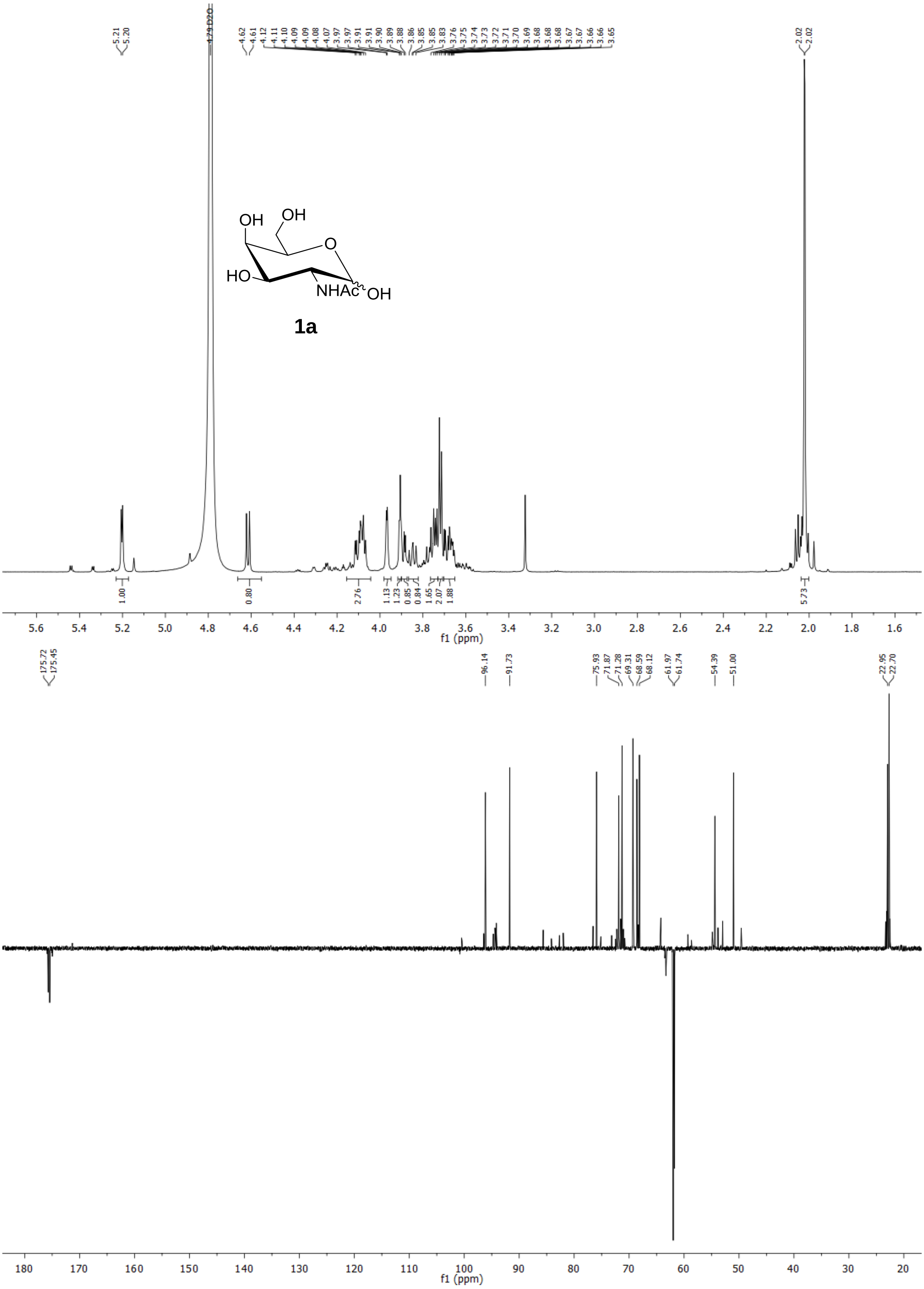
5.21
5.20
4.79 D2O
4.62
4.61
4.12
4.11
4.10
4.09
4.09
4.08
4.07
3.97
3.97
3.91
3.91
3.90
3.89
3.88
3.86
3.85
3.85
3.83
3.76
3.75
3.74
3.73
3.72
3.71
3.70
3.69
3.68
3.68
3.67
3.67
3.66
3.66
3.65
2.02
2.02
OH
OH
O
HO
NHAc
OH
1a
1.00
0.80
2.76
1.13
1.23
0.85
0.84
1.65
2.07
1.88
5.73
f1 (ppm)
175.72
175.45
96.14
91.73
75.93
71.87
71.28
69.31
68.59
68.12
61.97
61.74
54.39
51.00
22.95
22.70
f1 (ppm)

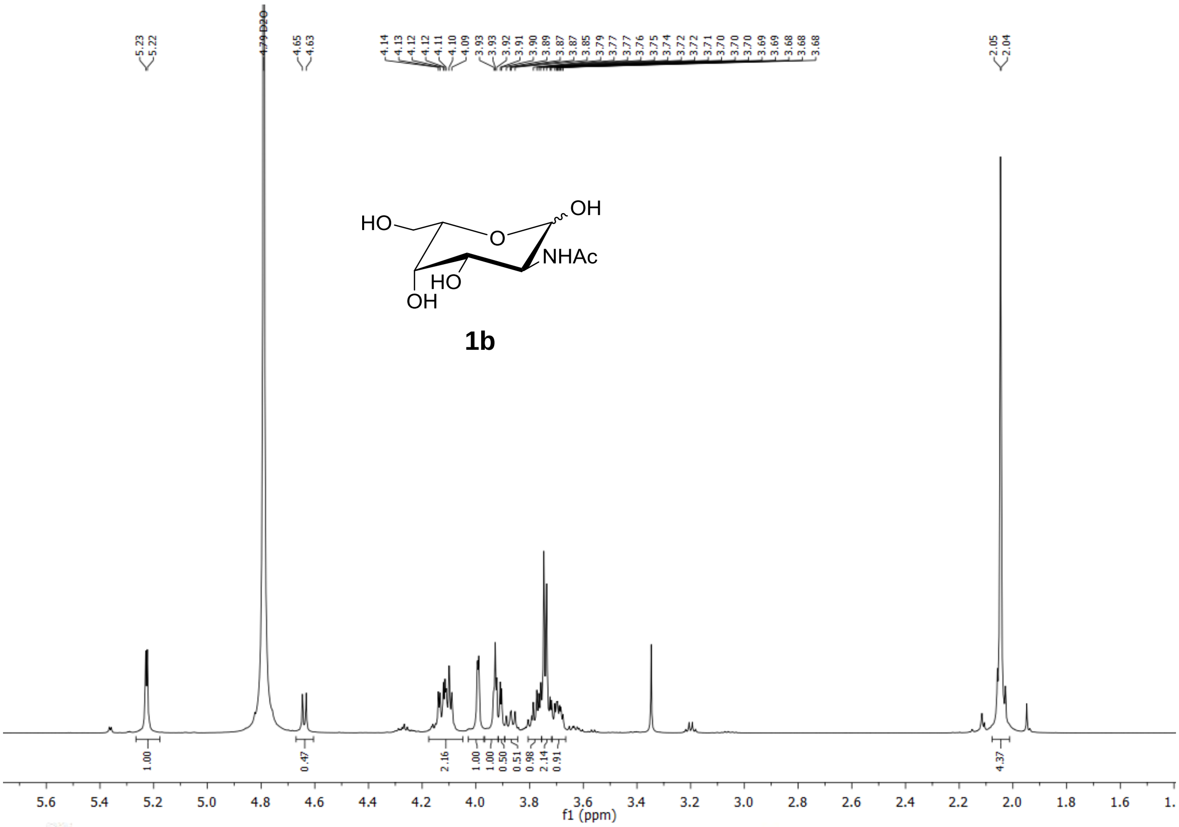
HO
O
OH
NHAc
HO
OH
1b
5.23
5.22
4.79 D2O
4.65
4.63
4.14
4.13
4.12
4.12
4.11
4.10
4.09
3.93
3.93
3.92
3.91
3.90
3.89
3.87
3.87
3.85
3.79
3.77
3.77
3.76
3.75
3.74
3.72
3.72
3.71
3.70
3.70
3.70
3.69
3.69
3.68
3.68
3.68
2.05
2.04
1.00
0.47
2.16
1.00
1.00
0.50
0.51
0.98
2.14
0.91
4.37
f1 (ppm)

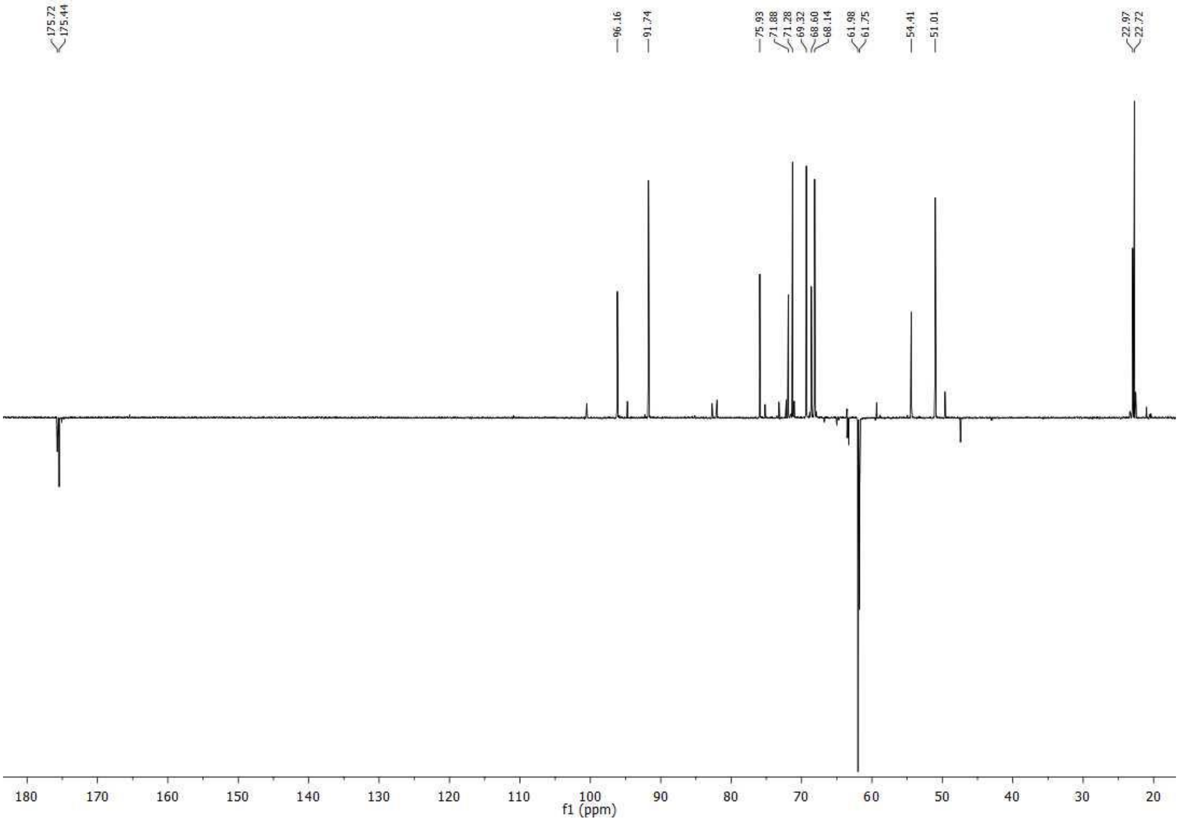
175.72
175.44
96.16
91.74
75.93
71.88
71.28
69.32
68.60
68.14
61.98
61.75
54.41
51.01
22.97
22.72
f1 (ppm)
